# Effect of Modified Vaccinia Ankara–5T4 and Low-Dose Cyclophosphamide on Antitumor Immunity in Metastatic Colorectal Cancer: A Randomized Clinical Trial

**DOI:** 10.1001/jamaoncol.2017.2579

**Published:** 2017-09-07

**Authors:** Martin Scurr, Tom Pembroke, Anja Bloom, David Roberts, Amanda Thomson, Kathryn Smart, Hayley Bridgeman, Richard Adams, Alison Brewster, Robert Jones, Sarah Gwynne, Daniel Blount, Richard Harrop, Melissa Wright, Robert Hills, Awen Gallimore, Andrew Godkin

**Affiliations:** 1 Division of Infection and Immunity, Cardiff University, Cardiff, Wales; 2 Velindre Cancer Centre, National Health Service (NHS) Trust, Cardiff, Wales; 3 South West Wales Cancer Centre, Singleton Hospital, NHS Trust, Swansea; 4 Oxford BioMedica, plc, Oxford, England; 5 Centre for Trials Research, Cardiff University, Cardiff, Wales; 6 Department of Gastroenterology and Hepatology, University Hospital of Wales, Cardiff

## Abstract

**Importance:**

The success of immunotherapy with checkpoint inhibitors is not replicated in most cases of colorectal cancer; therefore, different strategies are urgently required. The oncofetal antigen 5T4 is expressed in more than 90% of cases of metastatic colorectal cancer (mCRC). Preliminary data using modified vaccinia Ankara–5T4 (MVA-5T4) in mCRC demonstrated that it safely induced serologic and T-cell responses.

**Objective:**

To determine whether antitumor immunity in mCRC could be increased using MVA-5T4, metronomic low-dose cyclophosphamide, or a combination of both treatments.

**Design, Setting, and Participants:**

In this randomized clinical trial, 55 patients with inoperable mCRC and prior stable disease after standard chemotherapy were enrolled at a single center and randomized to watch and wait (n = 9), cyclophosphamide treatment only (n = 9), MVA-5T4 only (n = 19), and a combination of MVA-5T4 and cyclophosphamide (n = 18). Patients were enrolled and treated from July 9, 2012, through February 8, 2016, and follow-up was completed on December 13, 2016. Data were analyzed based on intention to treat.

**Interventions:**

Patients randomized to a cyclophosphamide group received 50 mg twice daily on treatment days 1 to 7 and 15 to 21. Patients randomized to a MVA-5T4 group received an intramuscular injection at a dose of 1 × 10^9^ 50% tissue culture infectious dose on treatment days 22, 36, 50, 64, 78, and 106.

**Main Outcomes and Measures:**

The predefined primary end point was the magnitude of anti-5T4 immune responses (5T4-specific T-cell and antibody levels) generated at treatment week 7. Secondary end points included analysis of the kinetics of anti-5T4 responses, progression-free survival (PFS), and overall survival (OS).

**Results:**

Fifty-two patients (38 men and 14 women; mean [SD] age, 64.2 [10.1] years) were included in the study analysis. The 5T4-specific antibody immune responses were significantly increased in the MVA-5T4 (83.41 [36.09] relative units [RU]; *P* = .02) and combination treatment (65.81 [16.68] RU; *P* = .002) groups compared with no treatment (20.09 [7.20] RU). Cyclophosphamide depleted regulatory T cells in 24 of 27 patients receiving MVA-5T4, independently prolonging PFS (5.0 vs 2.5 months; hazard ratio [HR], 0.48; 95% CI, 0.21-1.11; *P* = .09). MVA-5T4 doubled baseline anti-5T4 responses in 16 of 35 patients, resulting in significantly prolonged PFS (5.6 vs 2.4 months; HR, 0.21; 95% CI, 0.09-0.47; *P* < .001) and OS (20.0 vs 10.3 months; HR, 0.32; 95% CI, 0.14-0.74; *P* = .008). No grade 3 or 4 adverse events were observed.

**Conclusions and Relevance:**

This initial randomized clinical immunotherapy study demonstrates a significant survival benefit in mCRC. Prior depletion of regulatory T cells by cyclophosphamide did not increase immune responses generated by MVA-5T4 vaccination; however, cyclophosphamide and MVA-5T4 each independently induced beneficial antitumor immune responses, resulting in prolonged survival without toxic effects. Larger clinical trials are planned to further validate these data.

**Trial Registration:**

isrctn.org Identifier: ISRCTN54669986

## Key Points

**Question:**

Does low-dose cyclophosphamide, modified vaccinia Ankara–5T4, or a combination enhance antitumor immunity in patients with metastatic colorectal cancer?

**Findings:**

In this randomized clinical trial of 55 patients, all 3 treatment regimens improved antitumor immunologic responses, prolonging survival with no safety concerns. The addition of cyclophosphamide did not affect the number of patients who responded to modified vaccinia Ankara–5T4; however, survival outcomes improved in patients with metastatic colorectal cancer who received low-dose cyclophosphamide treatment.

**Meaning:**

These data support the importance of well-targeted antitumor immune responses, show the safety and antitumor activity of low-dose cyclophosphamide and modified vaccinia Ankara–5T4, and support further investigation in metastatic colorectal cancer.

## Teaser

This randomized clinical trial assesses the effect of modified vaccinia Ankara–5T4, metronomic low-dose cyclophosphamide, or a combination of both treatments on antitumor immunity in patients with metastatic colorectal cancer.

## Introduction

Colorectal cancer (CRC) is the second leading cause of death from cancer.^1^ Although early stages are often cured by surgical resection, the prognosis for patients with metastatic colorectal cancer (mCRC) is very poor, with a 5-year survival rate of 7%.^2^

An unmet need for improved therapies exists; although immunotherapy has been at the forefront of recent advances, results in CRC have been disappointing. More than 96% of patients with mCRC have microsatellite stable tumors^3^ that do not respond to current immunotherapies, possibly owing to decreased incidence of neoantigens.^4^ We hypothesized that in these patients, a well-targeted immune response against an up-regulated tumor antigen with minimal expression on healthy background tissues represents a potentially more effective therapy. One candidate is 5T4, a trophoblast glycoprotein with restricted expression to several human adenocarcinomas, including more than 90% of CRCs.^7^ Previous studies^9^ demonstrated that 5T4-specific interferon-γ–positive (IFN-γ^+^) T-cell responses correlate with tumor stage, providing protection against metastasis.

Herein, we sought to improve 5T4 immune responses in patients with mCRC through vaccination with an immunogenic, nonreplicating modified vaccinia Ankara–5T4 (MVA-5T4; TroVax; Oxford BioMedica, plc). This vaccine has demonstrated efficacy in preclinical models of colon cancer via the induction of humoral anti-5T4 responses.^11^ Early indications in mCRC demonstrated an excellent safety profile, with the induction of anti-5T4 responses correlating with disease control, thus warranting further studies in randomized clinical trials.^12^

Previous attempts at vaccination strategies targeting up-regulated tumor antigens have been largely unsuccessful for many tumor types; however, a recent trial of MVA-5T4 encoding mucin 1 in advanced non–small cell lung cancer demonstrated improvement in progression-free survival (PFS).^13^ Such vaccines work by inducing the intracellular expression of their respective transgene, allowing the tumor antigen to be processed by major histocompatibility complex classes I and II pathways. Given that activation of the adaptive immune response may concurrently stimulate tumor-specific regulatory T (Treg) cells, we also sought to test the hypothesis that the effectiveness of cancer vaccines is improved by prior administration of a Treg-depleting agent. In low doses, cyclophosphamide has demonstrated numerous immune-potentiating effects, including the depletion and reduced functionality of Tregs.^14^ However, to date, low-dose metronomic cyclophosphamide treatment has not been evaluated in a randomized clinical setting for cancer.

The trial reported herein is a randomized phase 1 and 2 clinical trial in patients with inoperable mCRC that assessed the effectiveness of cyclophosphamide in increasing the immunotherapeutic potential of MVA-5T4. We present the final analyses of primary and secondary end points, including an assessment of how anti-5T4 immune responses and Treg cell depletion are associated with patient survival.

## Methods

### Study Design and Participants

This open-label study was performed in a single center in the Clinical Research Facility, University Hospital of Wales, Cardiff. A copy of the trial protocol is found in Supplement 1. The Gene Therapy Advisory Committee and the Cardiff and Vale University Health Board ethics committee approved the study. Trial authorization was granted from the Medicines and Healthcare Products Regulatory Authority. This trial was conducted in compliance with International Conference on Harmonisation and Good Clinical Practice regulatory requirements. All patients gave written, informed consent before trial inclusion.

Patients underwent evaluation for recruitment at Velindre National Health Service Trust, Cardiff, and the South West Wales Cancer Centre, Singleton Hospital, Swansea. Patients were eligible if they had inoperable stage IV CRC and evidence of responding or stable disease within 4 weeks before trial entry. Previous findings^16^ indicate that patients receiving palliative chemotherapy can safely be given protracted breaks from chemotherapy with no evidence of a worsening of their outcome. However, patients with elevated platelet counts (>400 000/μL [to convert to /×10^9^/L, multiply by 1.0]) did not tolerate chemotherapy-free intervals and were excluded from this study. Additional exclusion criteria included hemoglobin levels less than 11 g/dL (to convert to grams per liter, multiply by 10.0), monocyte count greater than 80 000/μL (to convert to /×10^9^/L, multiply by 0.001), completion of first-line chemotherapy within 2 weeks before the start of treatment, clinically apparent autoimmune disease, or use of immunosuppressants. Key inclusion criteria included World Health Organization performance status of 0 to 2, lymphocyte count of at least 500/μL (to convert to /×10^9^/L multiply by 0.001), and neutrophil count of greater than 1200/μL (to convert to /×10^9^/L, multiply by 0.001).

### Randomization and Masking

**Figure 1.**
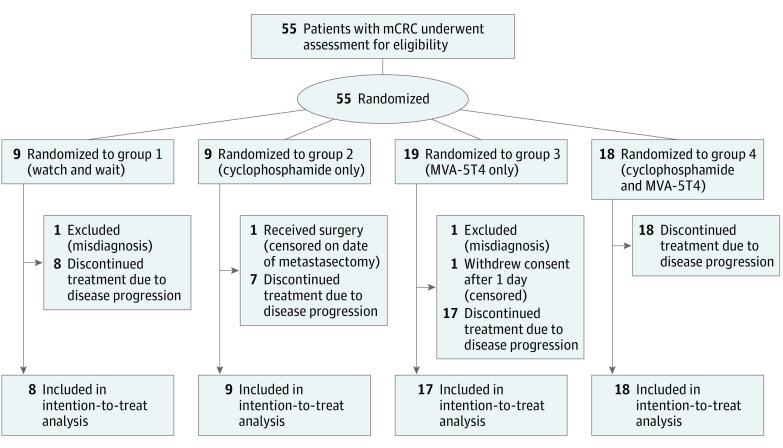
CONSORT Flow Diagram of Study Enrollment mCRC indicates metastatic colorectal cancer; MVA-5T4, modified vaccinia Ankara–5T4 (TroVax; Oxford Biomedica, plc).

The trial was based on a 2 × 2 factorial design. Patients were randomized 1:1 between receiving cyclophosphamide and not, and 2:1 between receiving MVA-5T4 and not, resulting in the following 4 treatment arms: control, or watch and wait (unless clinically indicated; group 1), metronomic cyclophosphamide only (group 2), MVA-5T4 only (group 3), or cyclophosphamide and MVA-5T4 (group 4) (Figure 1). Randomization was undertaken at the Clinical Trials Office, University Hospital of Wales, using an unstratified balanced block design, with the outcome communicated to the attending physician immediately after randomization and participant enrollment. Treatment allocation was not masked in this open-label study.

### Procedures

**Figure 2.**
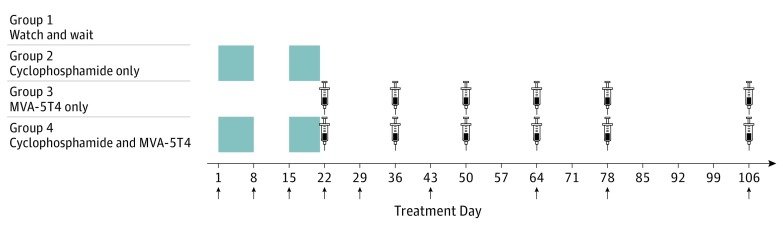
Trial Treatment Schedule Cyclophosphamide was given in a dose of 50 mg twice daily at treatment days 1 to 7 (week 1) and 15 to 21 (week 3) to groups 2 and 4 (shaded blocks). The modified vaccinia Ankara–5T4 (MVA-5T4; TroVax; Oxford Biomedica, plc) was given as an intramuscular injection in a dose of 1 × 10^9^ 50% tissue infectious dose at treatment days 22 (week 4), 36 (week 6), 50 (week 8), 64 (week 10), 78 (week 12), and 106 (week 16) to groups 3 and 4 (injection graphic). Arrows indicate days when blood samples were obtained.

Cyclophosphamide (Pharmacia Ltd) was orally administered in doses of 50 mg twice per day on treatment days 1 to 7 and 15 to 21 or until the patient experienced relapse. Groups 2 and 4 patients were contacted by telephone during cyclophosphamide treatment to ensure compliance. MVA-5T4 was administered in an intramuscular injection at a dose of 1 × 10^9^ 50% tissue culture infectious dose (TCID_50_) on treatment days 22, 36, 50, 64, 78, and 106. Peripheral blood samples were obtained at regular intervals (Figure 2).

We performed physical examinations and full blood cell counts, measurement of urea and electrolyte levels, and liver function tests at each blood sample obtainment. Tumor burden was assessed quantitatively using Response Evaluation Criteria in Solid Tumors (RECIST) criteria after computed tomographic scans of the abdomen and chest during treatment week 12. Beyond 16 weeks of treatment, assessments were performed every 12 weeks until documented disease progression, whereby the patient would be treated with standard chemotherapy as indicated.

To assess immunologic responses, peripheral blood mononuclear cells were isolated from heparinized blood samples by centrifugation over density gradient media (Ficoll; GE Healthcare) and cultured in triplicate with 5T4 peptide pools or control antigens for 14 days. We performed IFN-γ enzyme-linked immunospot assays to assess for 5T4-specific T-cell responses, as previously described.^10^ Positive responses were identified as having at least 20 spot-forming cells per 10^5^ cultured peripheral blood mononuclear cells and double the number of spots compared with background.

To perform T-cell counts, 3 μL of CD3-APC, CD4-PE, and CD8-PerCPCy5.5 were added to 50 μL of whole heparinized blood using a reverse pipetting technique. Red blood cells were lysed before addition of 50 μL absolute counting beads (CountBright; ThermoFisher); samples were acquired on a cell analyzer (FACSCanto II; BD Biosciences), and cell counts were calculated according to the manufacturer’s instructions. To calculate the proportion of CD4^+^ T cells expressing Foxp3, fresh peripheral blood mononuclear cells were stained with a fixable aqua dead cell stain kit (Live/Dead; Thermo Fisher Scientific) and then with CD4-APCh7, followed by fixation and/or permeabilization and intracellular staining with Foxp3-APC.

Plasma samples were collected from blood samples separated over the density gradient media to measure 5T4-specific antibody level, determined using semiquantitative enzyme-linked immunosorbent assays as previously described.^17^ Polyclonal plasma positive for 5T4 was used as a standard curve for each assay. A 2-fold increase in 5T4 antibody relative units was established as the level at which a false-positive rate of less than 1% could be expected, and antibody levels were considered to be positive above this value.

### Outcomes

Given the trial’s primary objective to measure the effect of MVA-5T4 and/or cyclophosphamide on antitumor immune responses, the primary end point was the magnitude of 5T4-specific responses at treatment day 43 (week 7). Secondary end points included the kinetics of anti-5T4 immune responses over time, PFS, overall survival (OS), treatment-emergent adverse events, and Treg cell depletion during cyclophosphamide treatment. We defined PFS as the time from the date of trial randomization to the date of first documented tumor progression, as determined by evidence of radiologic progression on computed tomographic scan or clinical deterioration as assessed by the oncologist. We defined OS as the time from the date of trial randomization to the date of death due to any cause.

To assess safety, we reported adverse event occurrences to the Data Monitoring and Ethics Committee after completion of treatment in every 6 patients in group 4. Safety in this group was assessed weekly for the first 4 weeks.

### Statistical Analysis

Follow-up was completed on December 13, 2016, by which all patients had experienced progression or received other treatments. Power was based on the mean effect size expected for each treatment, allowing for possible synergy between the 2 treatments. A randomization of 27 vs 27 patients to receive cyclophosphamide or not gave 80% power to detect a moderate difference of 0.8 SD in antitumor immune response or other laboratory markers. For MVA-5T4 randomization, a total of 54 patients (allocated as 36 vs 18) gave 80% power to detect a difference of 0.83 points between MVA-5T4 or not. Because the possible synergy between cyclophosphamide and MVA-5T4 was of interest, the MVA-5T4 randomization was in a ratio of 2:1; if such synergy was observed, a comparison of adding cyclophosphamide with MVA-5T4 would contain 36 patients, enough for a difference of 1.0 SD with 80% power.

All analyses were performed on an intention-to-treat basis, with patients ineligible for the trial excluded. Wilcoxon matched-pairs signed rank tests were used to compare nonparametric data sets, including the assessment of Treg cell depletion during cyclophosphamide treatment and 5T4 immune responses generated; patients were subdivided based on these results, according to end points stipulated in the trial protocol. We analyzed OS and PFS using log-rank tests and displayed using Kaplan-Meier plots. Categorical data analysis was performed using stratified Mantel-Haenszel tests. Effect sizes are displayed as Peto odds ratios with 95% CIs. Analyses of each treatment were performed stratified for the other treatment allocation within the factorial design, and stratified results were displayed as forest plots. *P* < .05 was considered to be statistically significant, and all tests of significance were 2 sided. Analyses were performed using SAS (version 9.4; SAS Institute, Inc) and GraphPad Prism (version 7; GraphPad Software, Inc) software.

## Results

From July 9, 2012, through February 8, 2016, 54 patients were recruited and randomized. One patient from group 3 withdrew consent before receiving the allocated intervention, and 1 patient each from groups 1 and 3 were later found to have undergone a curative procedure before enrollment (Figure 1). These 3 patients were not included in the analyses of immune responses, PFS, and OS; however, an additional patient was recruited to group 3 and was included in the analyses. Thus, 52 patients were eligible for evaluation (38 men and 14 women; mean [SD] age, 64.2 [10.1] years). The baseline characteristics of the patients randomized to the 4 groups are shown in eTable 1 in Supplement 2. All patients presented with liver, lung, and/or peritoneal metastases.

**Figure 3.**
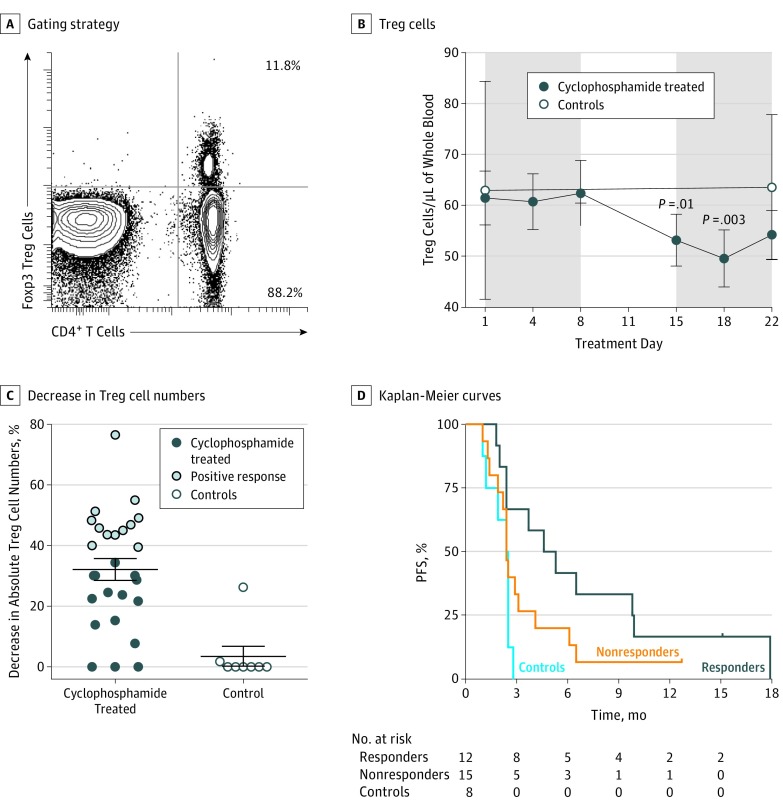
Progression-Free Survival (PFS) in Response to Cyclophosphamide Peripheral Foxp3^+^ regulatory T (Treg) cells were enumerated by initially counting the CD3^+^CD4^+^ T cells in whole blood, followed by phenotypic analysis to determine the proportion of CD4^+^ T cells expressing Foxp3. A, Example of the gating strategy. B, Overall Treg cell numbers are shown at indicated points during cyclophosphamide treatment in 27 cyclophosphamide-treated patients compared with 8 control individuals. C, The maximum percentage decrease in absolute CD4^+^Foxp3^+^ Treg cells from treatment days 4 to 22 compared with baseline was measured. The threshold for a positive response was set at the upper 95% CI of 39.4% Treg cell depletion, with 12 patients meeting this criterion. D, Kaplan-Meier survival curves are given for the 12 cyclophosphamide responders (>39.4% Treg cell depletion), the 15 cyclophosphamide nonresponders (≤39.4% Treg cell depletion), and 8 controls. The median PFS among cyclophosphamide responders was 5.0 months compared with 2.5 months for nonresponders and 2.5 months for controls (hazard ratio, 0.48; 95% CI, 0.21-1.11; *P* = .09).

The key objectives of the study were to determine the effect of low-dose cyclophosphamide on antitumor immune responses and whether such treatment could enhance immune responses generated by MVA-5T4 vaccination. Cyclophosphamide alone induced many immunologic perturbations, most evident being an increase in IFN-γ^+^ 5T4-specific T-cell responses and the depletion of Foxp3^+^ Treg cells (24 of 27 patients in groups 2 and 4) (Figure 3A); significant depletions in Treg cells were noted during treatment week 3 at days 15 (53.22/μL [5.08/μL]; *P* = .01) and 18 (49.63/μL [5.61/μL]; *P* = .003) compared with treatment day 1 (61.48/μL [5.28/μL]) (Figure 3B). With a threshold of a decrease in absolute Treg cell numbers (ie, percentage of Treg cell depletion) set above the upper 95% CI (Figure 3C), those patients exhibited prolonged median PFS compared with nonresponding (ie, below the threshold) patients (5.0 vs 2.5 months; hazard ratio [HR], 0.48; 95% CI, 0.21-1.11; *P* = .09) (Figure 3D) (data are shown stratified by MVA-5T4 in eFigure 1 in Supplement 2). Thus, effective cyclophosphamide-induced Foxp3^+^ Treg cell depletion was associated with prolonged PFS, but statistical significance was not reached likely owing to sample size and the study not being powered to directly address this. The Treg cell numbers returned to baseline by treatment day 29 in patients treated with cyclophosphamide only and remained at this level for the duration of the trial.

**Figure 4.**
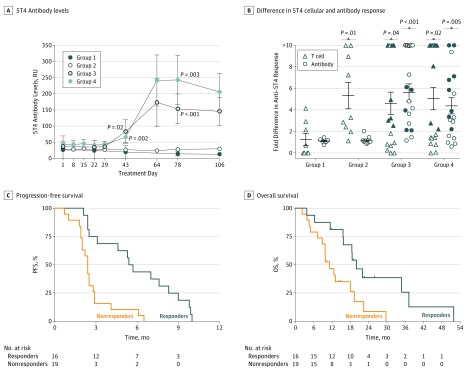
Anti-5T4 Immunologic Responses Associated With Survival A, 5T4-specific antibody levels were measured in plasma samples taken throughout the course of the trial. RU indicates relative unit. All *P* values are calculated compared with group 1. B, Fold increases in 5T4-specific interferon-γ–positive (IFN-γ^+^) T-cell and antibody responses were calculated by dividing the highest response to treatment at treatment days 8 through 106 by baseline (treatment day 1) level. Shaded symbols indicate the 16 recipients of the modified vaccinia Ankara–5T4 (TroVax; Oxford Biomedica, plc) (groups 3 and 4) demonstrating more than a 2-fold increase in anti-5T4 T-cell and antibody responses at any treatment day (responders). C and D, These patients exhibited significantly increased progression-free survival (PFS) (hazard ratio [HR], 0.21; 95% CI, 0.09-0.47; *P* < .001) and overall survival (OS) (HR, 0.32; 95% CI, 0.14-0.74; *P* = .008) compared with less than a 2-fold response to MVA-5T4 (nonresponders). Groups are described in Figure 1.

The presence of anti-5T4 immune responses was analyzed throughout the trial. Significant increases in 5T4 antibody level were evident after 2 vaccinations at treatment day 43 in group 3 (83.41 [36.09] relative units [RU]; *P* = .02) and group 4 (65.81 [16.68] RU; *P* = .002) (Figure 4A) compared with group 1 (20.09 [7.20] RU); thus, the primary end point was met. After treatment day 43, 5T4 antibody levels increased further for many patients, including at treatment day 78 in group 3 (153.6 [45.94] RU; *P* < .001) and group 4 (242.9 [76.69] RU; *P* = .003), compared with group 1 (14.41 [3.36] RU) (Figure 4A). Despite group 4 exhibiting larger increases compared with group 3 in anti-5T4 antibody titers (and corresponding anti-MVA titers in eFigure 2 in Supplement 2) at treatment days 64, 78, and 106, differences were not significant. Anti-MVA antibodies were detected at similar points, but only small nonsignificant reductions were noted in 5T4 antibody levels after treatment day 64. When analyzing secondary end points, development of 5T4 antibodies was consistent among MVA-5T4-treated patients, with 15 of 17 group 3 and 13 of 18 group 4 patients having more than a 2-fold increase in anti-5T4 antibody levels at some time during the trial (Figure 4B). A single instance was found of a cyclophosphamide-only treated patient also having a doubled anti-5T4 antibody response during treatment (Figure 4B).

The maximum increase in baseline 5T4-specific IFN-γ^+^ T-cell responses revealed varying degrees of T-cell response to MVA-5T4 vaccination, with 20 of 35 patients having more than a 2-fold increase. Among groups 1 and 2, 10 of 17 patients had more than a 2-fold increase, owing mostly to cyclophosphamide increasing anti-5T4 T-cell responses via Treg cell depletion (Figure 4B).

When considering all trial participants who had more than a 2-fold increase in anti-5T4 T-cell and antibody responses to cyclophosphamide or MVA-5T4 at any instance during the trial (Figure 4B), the increase was associated with prolonged PFS (5.7 vs 2.4 months; HR, 0.58; 95% CI, 0.31-1.09; *P* = .09) (eFigure 3A in Supplement 2) and OS (20.0 vs 13.1 months; HR, 0.56; 95% CI, 0.28-1.12; *P* = .10) (eFigure 3B in Supplement 2). Among groups 3 and 4 MVA-5T4–treated patients, this effect became more apparent because removing groups 1 and 2 patients from the analysis revealed a significant difference in PFS (5.6 vs 2.4 months; HR, 0.21; 95% CI, 0.09-0.47; *P* < .001) (Figure 4C) and OS (20.0 vs 10.3 months; HR, 0.32; 95% CI, 0.14-0.74; *P* = .008) (Figure 4D). Large increases in MVA titers to MVA-5T4 were also significantly associated with PFS (HR, 0.26; 95% CI, 0.11-0.59; *P* = .001) (eFigure 2B and C in Supplement 2) but not OS (HR, 0.69; 95% CI, 0.32-1.51; *P* = .36) (eFigure 2B and D in Supplement 2); thus, general immunologic responsiveness of the patient may also determine outcome.

**Figure 5.**
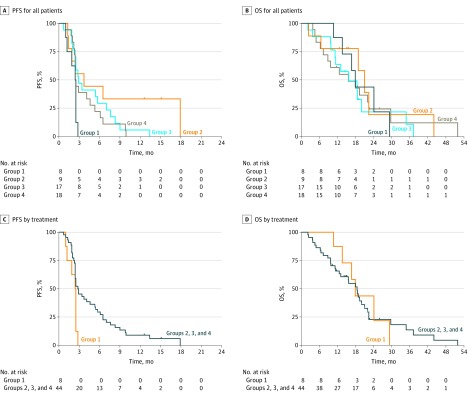
Kaplan-Meier Survival Curves by Trial Group Outcomes for all patients by trial group are indicated for progression-free survival (PFS) and overall survival (OS). Outcomes of 44 patients receiving any treatment (groups 2, 3, and 4) vs 8 control individuals (group 1) are indicated for PFS (HR, 0.41; 95% CI, 0.20-0.88; *P* = .02) and OS (HR, 1.03; 95% CI, 0.43-2.47; *P* = .95). Groups are described in Figure 1.

Although beneficial immunologic responses to cyclophosphamide or MVA-5T4 were independently associated with prolonged PFS compared with no treatment (group 1), neither treatment was more effective than the other (eFigure 1 and eFigure 4A and B in Supplement 2), nor did patients receiving combination treatment in group 4 exhibit improved survival compared with MVA-5T4–only group 3 (Figure 5A and B). Seven of 18 patients in group 4 had 5T4 T-cell and antibody responses at any point in the trial, but this was not statistically different from the 9 of 17 patients in group 3 who had similar responses to MVA-5T4 alone (*P* = .60) (eFigure 1A in Supplement 2). Therefore, the addition of cyclophosphamide does not enhance the effectiveness of MVA-5T4, nor does it appear to lessen response to vaccination.

All 3 treatment groups demonstrated improved PFS but not OS compared with the control group (Figure 5C and D); OS data are difficult to interpret because subsequent interventions offered to these patients is beyond our control. Patients in group 2 demonstrated the greatest increase in median PFS compared with group 1 (HR, 0.32; 95% CI, 0.09-1.09; *P* = .07) (Figure 5A), although the number of patients is small. Therefore, in patients with mCRC, receiving treatment with MVA-5T4 or low-dose cyclophosphamide was more effective than allowing a protracted break from chemotherapy. In addition, we found no instances of treatment-related grade 3 or 4 adverse events (eTable 2 in Supplement 2), suggesting an excellent safety profile of these interventions.

## Discussion

This randomized study demonstrated the clinical benefit of a combined vaccine and low-dose cyclophosphamide in the treatment of mCRC. Each treatment improved antitumor immunity through different mechanisms; cyclophosphamide was effective at depleting Foxp3^+^ Treg cells, resulting in increased anti-5T4 T-cell responses, which is associated with survival, and MVA-5T4 was effective at inducing cellular and humoral anti-5T4 responses, increasing PFS and OS in a subgroup analysis of patients with and without a treatment response. In combination, however, cyclophosphamide did not improve anti-5T4 responses during MVA-5T4 treatment despite a modest increase in anti-5T4 antibody level. This finding may reflect cyclophosphamide blocking the priming and proliferation of other important immune cell subsets required for effective vaccination (eg, tumor antigen–specific effector T cells and dendritic cells).^18^ Future studies will aim to decipher the exact effect of metronomic oral cyclophosphamide on immunologic responses and how it might impinge on other cell populations.

Saito et al^20^ recently hypothesized that depletion of suppression-competent Treg cells expressing high levels of Foxp3 and abundant in CRC may provide clinical benefit, and our trial findings appear to corroborate their hypothesis. Several studies^21^ have indicated a better prognosis when tumors are infiltrated with relatively high numbers of Foxp3^+^ Treg cells. A previous study^10^ demonstrated that as tumors advance, peripheral Treg cell proportion and suppressive capacity increase; in addition, Treg cell depletion significantly improves antitumor immune responses in mice^24^ and patients with CRC.^9^ In the present trial, when Treg cells were most effectively depleted, median PFS doubled (from 2.5 to 5.0 months). Although our evidence is limited to depletion of peripherally derived Foxp3^+^ Treg cells, the increase in PFS indicates effective tumor control through the probable concurrent depletion of intratumoral Treg cells, thus removing the suppression of intratumoral effectors. We find it plausible that colorectal tumor–specific Treg cells in advanced disease are detrimental to patients, and high preexisting Foxp3^+^ Treg cell infiltration is a bystander effect of a larger antitumor immune response.^26^

Regardless of mechanism, anti-5T4 responses during cyclophosphamide treatment or MVA-5T4 vaccination were associated with a statistically significant increase in PFS and OS. Similar to previous trials of MVA-5T4, in which intramuscular injection of 1 × 10^9^ TCID_50_ of MVA-5T4 induced the strongest immune response correlating with disease control,^17^ the vaccine was shown to be safe and well tolerated, and the induction of an anti-5T4 immune response did not result in any detrimental off-target autoimmunity.

Prior chemotherapeutic regimens in this group of patients with mCRC appeared to have little effect on responses to either immunotherapeutic treatment (eTable 1 in Supplement 2), although some patients began the trial with relatively high preexisting anti-5T4 responses, potentially induced by prior treatments. We found no evidence of preexisting responses correlating with improved outcomes, although patients with higher preexisting 5T4 antibody levels exhibited significantly higher 5T4 antibody responses to MVA-5T4 during treatment.

Large increases in Treg cells occurred in several patients regardless of prior cyclophosphamide treatment, in particular among patients with an HLA-DR1^+^/DQ5^+^ response after a single MVA-5T4 injection. Although this factor, along with increased MVA antibody level (eFigure 2 in Supplement 2), initially suggested the induction of peripheral tolerance to MVA-5T4, anti-5T4 T-cell and antibody responses remained largely unaffected. We also found no correlation between high anti-MVA titers and low anti-5T4 responses, although an association was identified between large increases in anti-MVA titers and PFS but not OS (eFigure 2 in Supplement 2), indicating that general patient health and immunocompetence may play a role in responsiveness to immunotherapy. Given that T-cell responses to the control antigen tuberculin purified protein derivative varied little during the trial and were not associated with patient outcome (eFigure 5 in Supplement 2), the key immune responses may be those generated against tumor antigens (eg, 5T4). This evidence supports exploring the use of MVA-5T4 (and other cancer vaccines^29^) earlier in the disease course.

### Limitations

Important limitations include the relatively small sample size, in particular when analyzing Treg cell depletion and performing survival analyses among group 2 patients receiving cyclophosphamide. These findings need to be validated in larger clinical trials. In addition, the overall survival readouts presented herein are hindered by subsequent treatments the patients receive beyond our control.

## Conclusions

This randomized clinical trial identified a subset of immunotherapy-responsive patients with mCRC who demonstrated better tumor control when given cyclophosphamide or MVA-5T4. Although cyclophosphamide failed to enhance MVA-5T4 immunogenicity, survival benefits with minimal adverse effects were demonstrated, and further investigation is warranted. Because of cyclophosphamide’s ineffectiveness in sustained Treg cell depletion during MVA-5T4 vaccination, we would propose the combination of MVA-5T4 with more potent blockade of tumor-derived immunosuppression for future development, for example with anti–CTLA-4 to eliminate intratumoral Treg cells^30^ or with anti–LAG-3 checkpoint inhibitors, given the extent of infiltration of highly suppressive LAG-3^+^CD4^+^ tumor-infiltrating T cells.^31^

## Notes

Supplement 1. Trial Protocol.

Supplement 2. **eTable 1.** Patient Baseline Characteristics **eTable 2.** Adverse Events **eFigure 1.** Effects of CPM and TroVax **eFigure 2.** Anti-MVA Antibody Responses Associated With Survival **eFigure 3.** Induced anti-5T4 Immunologic Responses Associated With Survival **eFigure 4.** PFS of TroVax-Treated or CPM-Treated Trial Groups **eFigure 5.** Tuberculin-PPD T-cell Response
