# Supplementary material for: Effect of Modified Vaccinia Ankara–5T4 and Low-Dose Cyclophosphamide on Antitumor Immunity in Metastatic Colorectal Cancer: A Randomized Clinical Trial
Source: JAMA Oncol. 2017 Sep 7;3(10):e172579. doi: 10.1001/jamaoncol.2017.2579 (PMC5824319; doi:10.1001/jamaoncol.2017.2579)
Supplement: Supplement 1. — Trial Protocol. [file jamaoncol-3-524-s001.pdf]

A PILOT STUDY TO ASSESS THE EFFECT OF REGULATORY T CELL DEPLETION ON 5T4-CONTAINING MVA (TROVAX®) VACCINATION IN PATIENTS WITH INOPERABLE METASTATIC COLORECTAL CANCER

TaCTiCC

TroVax® and Cyclophosphamide Treatment in Colorectal Cancer

Protocol version number and date:	V 5 November 2012	
Sponsor:	Cardiff University	
Trial Identifiers:	EudraCT number:	2010-024380-41
	ISRCTN:	ISRCTN54669986
	Protocol Number:	SPON868-10
	GTAC Number:	GTAC 175

CARDIFF UNIVERSITY PRIFYSGOL CAERDYDD

ECMC

TRIAL MANAGEMENT GROUP

Chief Investigator	Clinical Collaborators	
Dr Andrew GodkinAddress; Institute of Infection & Immunity,<br>Henry Wellcome Building, Heath Park,<br>Cardiff CF14 4XN<br>Telephone: 02920 687304 / 029 206 87003<br>Email:godkinaj@cf.ac.uk	Dr Robert Jones<br>(co-investigator and lead clinical oncologist)<br>Address: Velindre Hospital, Whitchurch,<br>Cardiff,<br>CF14 2TL<br>Telephone: 029 2061 6560<br>Email: Jonesrh7@cardiff.ac.uk	Dr Richard Adams<br>Address: Velindre Hospital, Whitchurch,<br>Cardiff, CF14 2TL<br>Telephone: 029 2061 5888 ext 6206<br>E-mail: AdamsRA3@cf.ac.uk
**Clinical Research Fellow**	**Research Fellow**	
Dr Thomas Pembroke<br>Address: Institute of Infection & Immunity,<br>Henry Wellcome Building,<br>Heath Park,<br>Cardiff CF14 4XN<br>Email: PembrokeT@cf.ac.uk	Martin Scurr<br>Address; Institute of Infection & Immunity,<br>Henry Wellcome Building, Heath Park,<br>Cardiff CF14 4XN<br>Email:scurrmj@cardiff.ac.uk	
**Statistics & Data Management**		
Dr Robert Hills<br>Address: Department of Haematology, Cardiff University School of Medicine, Cardiff CF14 4XN<br>Telephone: 02920 744647<br>Fax: 029 2047 4655<br>Email: hillsrk@cf.ac.uk	Dr Awen Gallimore<br>Address: Institute of Infection & Immunity,<br>Henry Wellcome Building, Heath Park, Cardiff CF14 4XN<br>Telephone: 02920 687012<br>Email: gallimoream@cf.ac.uk	

Pharmacy	Operational	Trial Management
Kathryn Bethune (pharmacist)<br>Address: Pharmacy Department, University Hospital of Wales, Heath Park, Cardiff CF14 4XW<br>Telephone: 02920 742991<br>Fax: 02920 744375<br>E-mail: Kathryn.Bethune@wales.nhs.uk	Dr Kathrine Craig<br>(operational director of the Clinical Research Facility)<br>Address: Clinical Research Facility. Upper Ground Floor C Block, University Hospital of Wales, Heath Park, Cardiff CF14 4XW<br>Telephone: 02920 748251<br>E-mail: craigkj@cardiff.ac.uk	Helen Clark (trial manager)<br>Address: Cancer Trials Unit. TB2 Room 20, University Hospital of Wales, Cardiff University, Heath Park, Cardiff, CF14 4XN<br>Telephone: 02920 745397<br>Fax: 02920 742289<br>Email: clarkh3@cf.ac.uk
**Other clinical collaborators**		
Dr Seema Arif<br>Address: Velindre Hospital, Whitchurch, Cardiff, CF14 2TL<br>Telephone: 029 2061 5888 ext 6184<br>E-mail: seema.arif@velindre-tr.wales.nhs.uk	Dr Tom Crosby<br>Address: Velindre Hospital, Whitchurch, Cardiff, CF14 2TL<br>Telephone: 029 2061 5888 ext 6202<br>E-mail: tom.crosby@velindre-tr.wales.nhs.uk	Dr Alison Brewster<br>Address: Velindre Hospital, Whitchurch, Cardiff, CF14 2TL<br>Telephone: 029 2061 5888 ext 6220<br>E-mail: Alison.brewster@velindre-tr.wales.nhs.uk

KEY CONTACTS

Randomisations:

HCTU Trials Office: 02920 74 8976/5397

Eligibility queries:

Dr Andrew Godkin: 02920 687304/687003/744879

Dr Robert Jones: 02920 616560

Dr Richard Adams 02920 615888 ext 6206

Clinical queries:

Dr Andrew Godkin: 02920 687304/687003/744879, or

Dr Robert Jones: 02920 616560

All Other queries:

HCTU Trials Office: 02920 74 8976/5397

TRIAL SUMMARY

Rationale

Colorectal cancer (CRC) is the third most common malignant disease in Western Europe. In the Cardiff and Vale University Health Board alone, 250 cases present each year. The current care of patients revolves around excision of the tumour, staging, and adjuvant 5-fluorouracil (5FU) or capecitabine based treatment for locally spreading disease, or as palliative chemotherapy for patients with advanced metastatic disease. Despite these advances, colorectal cancer remains the second leading cause of death from cancer in Wales. Chemotherapy has a significant morbidity and mortality associated with its use. We aim to build on our research to develop less toxic immunoadjuvants to treat patients.

It is now accepted that immune responses can be directed to antigens expressed in tumours (tumour-associated antigens or TAA) and control or kill the tumour. Our research in Cardiff indicates that CRC drives the expansion of a specific regulatory T cell (Treg) population which controls anti-tumour immune responses to the detriment of the patient. We have found that patients have an increased frequency of Tregs compared to controls, and that these Tregs specifically inhibited anti-tumour immune responses directed to the TAAs e.g.5T4 (an oncofetal antigen).

Methods

This study will assess the efficacy of using either cyclophosphamide, or a pox virus based vaccine containing the tumour antigen 5T4 called TroVax® (Oxford BioMedica), or both, in a 2x2 factorial design to deplete T-regs and enhance an immune response following completion of an initial 12 weeks of palliative chemotherapy. Patients who have inoperable metastatic disease will be recruited.

Blood samples will be obtained to measure anti-tumour immune responses and

lymphocyte subset phenotype in the laboratory using established techniques.

Although the study is not powered to measure a difference in clinical outcome, clinical parameters will be recorded to try to establish a correlation between immune responses and clinical outcome.

Primary Objective

i) to measure the effect of treatment with TroVax® and/or cyclophosphamide on anti-tumour immune responses in patients with colorectal cancer

Secondary Objectives

i) to investigate whether there is an improvement in Overall Survival (OS) in the patients treated with TroVax® and/or cyclophosphamide versus not receiving the experimental therapy

ii) to investigate whether Time To Progression (TTP)[1] with death as a competing risk differs between the TroVax® and/or cyclophosphamide versus not receiving the experimental therapy

iii) to investigate whether Progression Free Survival (PFS)[2] differs between the TroVax® and/or cyclophosphamide versus not receiving the experimental therapy

iv) to compare adverse events, laboratory measurements and vital sign measurements among the treatment groups

v) to compare the efficacy of Treg depletion in the different groups

vi) to explore the relationships between immune response (antibody and cellular responses against the tumour antigen 5T4 and the MVA viral vector) and clinical response (progression free survival, overall survival, time to progression, tumour

[1] TTP: time to disease progression from entering the trial (exclude death from non-tumour causes)
[2] PFS: time to disease progression or death from any cause from entering the trial with censoring for patients lost to follow up

response)

<u>Primary endpoint</u>

i) the magnitude of anti-tumour (5T4) immune responses at week 7

<u>Secondary endpoints</u>

i) the kinetics of anti-tumour (5T4) immune responses

ii) Overall Survival

iii) Time To Progression

iv) Progression Free Survival

v) incidence, nature, severity, relatedness and seriousness of treatment-emergent adverse events and clinically significant abnormal laboratory results

vi) T-reg depletion at week 4

Contents

TRIAL MANAGEMENT GROUP.. 2

KEY CONTACTS.. 3

TRIAL SUMMARY.. 4

1 Background & Rationale.. 9
2 TRIAL OBJECTIVES, ENDPOINTS & DESIGN.. 13
2.1 Objectives.. 13
2.1.1. Primary Objective.. 13

2.1.2. Secondary Objectives.. 13

2.2 Endpoints.. 14
2.2.1 Primary Endpoint.. 14

2.2.2 Secondary Endpoints.. 14

2.3 Trial Design.. 14
2.4 Trial Flowchart (weeks 1-8).. 16
3 TRIAL MEDICATION.. 18
3.1 Investigational Medicinal Product.. 18
3.2 Dosing Regimen.. 19
3.3 Drug accountability.. 20
3.4 Subject compliance.. 20
3.5 Concomitant medication.. 20
4 SELECTION & WITHDRAWAL OF TRIAL SUBJECTS.. 21
4.1 Inclusion criteria.. 21
4.2 Exclusion criteria.. 21
4.3 Selection of participants.. 23
4.4 Randomisation procedure.. 23
4.5 Withdrawal of subjects.. 24
4.6 Expected duration of trial.. 24
5 TRIAL PROCEDURES.. 25
5.1 By visit.. 25
5.2 Sample collection.. 25
5.3 Laboratory tests.. 26
6 ASSESSMENT OF EFFICACY.. 26
6.1 Primary efficacy parameters.. 26
6.2 Secondary efficacy parameters.. 26
6.3 Procedures for assessing / measuring efficacy parameters.. 27
6.3.1 Primary efficacy parameters.. 27

6.3.2 Secondary efficacy parameters.. 28

7 ASSESSMENT OF SAFETY.. 29
7.1 Specification, timing and recording of safety parameters.. 29
7.2 Adverse event reporting.. 29
7.2.1 Definitions 29

7.2.2 Reporting responsibilities.. 31

7.3 Treatment stopping rules.. 33
8 STATISTICS.. 33
8.1 Sample size.. 34
8.2 Randomisation.. 35
8.3 Analysis.. 35
9 DATA MONITORING & ETHICS COMMITTEE.. 35
10 DIRECT ACCESS TO SOURCE DATA AND DOCUMENTS.. 36
11 ETHICS & REGULATORY APPROVALS.. 36
12 QUALITY ASSURANCE.. 37

13 DATA HANDLING 37
14 PUBLICATION POLICY 38
15 FINANCIAL ASPECTS AND INSURANCE 38
16 REFERENCES 39
17 SIGNATORIES 41
APPENDIX 1 Preparation, Administration and Handling of Investigational Medicinal Products 42
APPENDIX 2 Instructions for the Handling, Storage, Reconstitution and Disposal of TroVax® 46
APPENDIX 3 WHO Performance Status 49

1 Background & Rationale

Colorectal cancer (CRC) is the third most common malignant disease in Western Europe. In the Cardiff and Vale University Health Board alone, 250 cases present each year. The current care of patients revolves around excision of the tumour, staging, and adjuvant 5-fluorouracil (5FU) or capecitabine based treatment for locally spreading disease, or as palliative chemotherapy for patients with advanced metastatic disease. Chemotherapy has evolved over the last 12 years from 5FU as a sole infusional agent to a combination of 5FU or its oral pro-drugs with newer classes of drugs such as irinotecan, oxaliplatin, bevacizumab and cetuximab.

Despite these advances, colorectal cancer remains the second leading cause of death from cancer in Wales. Chemotherapy has a significant morbidity and mortality attached to its use, and overall the long term results of this form of treatment are obviously very disappointing. We aim to build on our research outlined below to develop less toxic immunoadjuvants to treat patients.

There is overwhelming evidence that immune responses can control and eradicate cancers. A large body of recent work from many different groups has demonstrated an increase in spontaneous and carcinogen-induced tumours in immunocompromised mice [1]. Epidemiological studies show that there is a greater relative risk compared to the general population for tumours not only associated with viral infections, but of non-viral origin (including breast, lung, pancreas, colon, renal tract and skin tumours) in individuals being treated with immunosuppressants [2].

Multiple studies have demonstrated a better prognosis in CRC patients if the numbers of tumour infiltrating lymphocytes (TILs) is high [3]. A recent study demonstrated the remarkable observation that the degree of cytotoxic TILs in CRC was the most important factor in prognosis, irrespective of the clinicopathological staging [4].

It is now accepted that immune responses can be directed to antigens expressed in tumours (tumour-associated antigens or TAA) and control or kill the tumour. These

anti-tumour responses can be adoptively transferred between animals by transferring lymphocytes from a protected to a naive animal or, in the case of humans, by expanding anti-tumour cells in vitro and infusing them back into the patient [5]. Anti-tumour immune responses may involve all branches of the immune response but the development of a robust effector CD4+ T cell response appears to be essential to allow an overall broad anti-tumour immune response to develop e.g. cytotoxic and antibody responses. Recently it has been demonstrated in humans that infusing anti-tumour CD4+ T cells allowed just such broad response to develop and destroy a solid tumour [6]. Individually tailored approaches are logistically impractical on a population basis and hence our interest is in developing widely applicable strategies.

Our research in Cardiff indicates that CRC drives the expansion of a specific regulatory T cell population that controls anti-tumour immune responses to the detriment of the patient.

The increasing success of immunotherapy for diseases such as melanoma, renal cell cancer, and breast cancer reflects a better understanding of factors involved in preventing anti-tumour immune responses. Much interest has recently been generated in the role of a naturally occurring population of $CD4^{+}CD25^{+}$ regulatory T cells (Tregs) characterised by expression of the transcription factor FoxP3, in controlling self-antigen specific responses in the periphery, and in controlling / preventing anti-tumour immune responses [7]. Dr Godkin, running a laboratory in partnership with another Cardiff University Reader with a Wellcome Trust University Award, Dr Gallimore, has had an interest in Tregs for several years. Dr Gallimore first demonstrated in murine models that depletion of Tregs can abrogate immunological non-responsiveness to syngeneic tumours *in vivo*, and allow a striking and complete protection of the animal following further tumour challenges [8]. Following these experiments, we have recently demonstrated that depletion of Tregs significantly delays or prevents the development of tumours using a carcinogen-induced tumour model in mice [9].

Results arising from murine models led us to explore the role of Tregs in patients with colorectal cancer. Several studies have observed an increased frequency of Tregs in

the peripheral blood of patients with lung, breast, stomach, pancreas and ovarian malignancies [5]. The presence of Tregs mixed in with the TILs of ovarian cancer has been associated independently with a poorer prognosis [10]. We have published the early results of a CRC study which demonstrated that patients had an increased frequency of Tregs compared to controls, and that these Tregs specifically inhibited anti-tumour immune responses directed to the TAAs 5T4 (an oncofetal antigen) and CEA [11].

Examination of the composition of TILs in resected advanced CRC from 41 subjects demonstrated a striking infiltrate of Tregs (Betts et al submitted). We have now examined changes in Tregs and anti-tumour immune responses in the peripheral blood of 62 patients undergoing resection of colorectal cancer and followed up for 12 months post-operatively. The findings suggest that the physical presence of CRC drives the development of a phenotypically distinct Treg population that inhibits anti-tumour responses (Betts et al submitted); to summarise:

- Tregs from patients express significantly higher levels of FoxP3 per cell compared to controls, as well as increased levels of the integrin CD49d; these increases *revert to normal* after the tumour is removed.
- The frequency of Tregs in blood falls after surgery but returns in subjects who develop disease recurrences/metastases.
- Approximately 60% of patients pre-operatively demonstrate anti-CEA or 5T4 $CD4^+$ T cell responses but only after Treg depletion *in vitro.*
- Post-operatively 90% of patients demonstrate higher frequency $CD4^+$ T cell responses and the level of Treg-mediated suppression falls unless the tumour recurs.
- In the group with tumour recurrence at 12 months, 100% of pre-operative measured anti-tumour immune responses were suppressed by Tregs, compared to those with no tumour recurrence at 12 months.

Considering the striking protection and enhanced anti-tumour immune responses we see in mice when Tregs are depleted, it seems highly plausible that Tregs in CRC,

certainly in advanced disease, are detrimental to patients.

Our results provide the rationale for this study: reducing the influence of Tregs in patients with CRC will augment anti-tumour responses and be to the benefit of patients; this project will explore the means of manipulating the Treg population in vivo combined with a 5T4 containing vaccine.

The immunogenicity of pox viruses has been recognised for over a hundred years, and they have been seen as an attractive vector to vaccinate proteins into a host [12]. TroVax® (OxfordBiomedica) uses a modified vaccina Ankara virus (MVA) which is attenuated and unable to replicate in the human host cell, yet is still highly immunogenic. It has already been given to cancer patients to induce immune responses to the 5T4 protein tumour antigen, and has emerged as a promising new vaccine candidate [13].

There have been 4 published phase I/II trials where TroVax® was given to patients with CRC [14]. Minimal side effects were recorded in doses up to 1 x 10^9 $TCID_{50}$. The most recent study published on TroVax® given to 365 renal cell cancer patients used 1 x 10^9 $TCID_{50}$ with minimal side effects / toxicity [15]. The fact that this dose is well tolerated and safe provides the rationale for setting the dose level in this trial. TroVax® has been shown to induce both T cell and B cell responses. The serological response to TroVax® indicated by anti-5T4 antibodies has been associated with better hazard ratio in several phase I/II trials [14]. The mechanism of effect is unknown. Antibodies may reflect a better T cell response, or may actually be effector molecules in killing cancer cells.

Cyclophosphamide is a well-established anti-proliferative agent. It was first shown to deplete regulatory T cells in murine models in the 1980s. However it has only recently been shown in humans that low dose cyclophosphamide depletes Tregs and restores T cell effector function [16, 17]. We envisage risks to be minimal: cyclophosphamide has been used extensively used for decades and its side effect profile is well

described (it has marketing authorisation and a summary of products characteristics (SmPC)). In this study we are using a 50mg bd metronomic dose.

Current treatment for the type of patients we will enrol in this trial is “watch and wait”, following an initial period of palliative chemotherapy to which they have either responded or had stable disease. This study will assess the efficacy of using either cyclophosphamide, or TroVax®, or both, in a 2x2 factorial design.

This study will be conducted to ICH-GCP regulatory requirements.

2 TRIAL OBJECTIVES, ENDPOINTS & DESIGN

2.1 Objectives

2.1.1. Primary Objective

i) to measure the effect of treatment with TroVax® and/or cyclophosphamide on anti-tumour immune responses in patients with colorectal cancer.

2.1.2. Secondary Objectives

i) to investigate whether there is an improvement in Overall Survival (OS) in the patients treated with TroVax® and/or cyclophosphamide versus not receiving the experimental therapy

ii) to investigate whether Time To Progression (TTP) with death as a competing risk differs between the TroVax® and/or cyclophosphamide versus not receiving the experimental therapy

iii) to investigate whether Progression Free Survival (PFS) differs between the TroVax® and/or cyclophosphamide versus not receiving the experimental therapy

iv) to compare adverse events, laboratory measurements and vital sign measurements among the treatment groups

v) to compare the efficacy of Treg depletion in the different groups

vi) to explore the relationships between immune response (antibody and cellular responses against the tumour antigen 5T4 and the MVA viral vector) and clinical response (progression free survival, overall survival, time to progression, tumour response)

2.2 Endpoints

2.2.1 Primary Endpoint

i) the magnitude of anti-tumour (5T4) immune responses at week 7

2.2.2 Secondary Endpoints

i) the kinetics of anti-tumour (5T4) immune responses

ii) Overall Survival

iii) Time To Progression

iv) Progression Free Survival

v) incidence, nature, severity, relatedness and seriousness of treatment-emergent adverse events and clinically significant abnormal laboratory results

vi) T-reg depletion at week 4

2.3 Trial Design

This study will be a randomised open label trial with a 2x2 factorial design, in patients with inoperable metastatic Colorectal Cancer. This study in patients with advanced disease will allow us to gather the essential preliminary information on safety, acceptability, logistic feasibility, and efficacy to help plan and apply for funding of down-stream phase II/III clinical trials.

It has been found that patients who are receiving palliative chemotherapy can safely be given protracted breaks i.e. chemotherapy "holidays" with no evidence of a worsening of their outcome [18]. However, those patients with elevated platelets at

start of chemotherapy did not tolerate chemotherapy-free intervals and therefore they will be excluded from this study [19].

Patients with stable or responding disease as defined by RECIST (Response Evaluation Criteria In Solid Tumours) following 12 weeks standard treatment will be recruited from clinic, and randomised to one of four groups:

Group 1: Watch and wait, i.e. no additional treatment unless clinically indicated.
Group 2: Metronomic cyclophosphamide.
Group 3: Vaccination (i.m.) TroVax®.
Group 4: Metronomic cyclophosphamide 50mg bd followed by TroVax®.

9 patients will be allocated to group 1, 9 patients will be allocated to group 2
18 patients will be allocated to group 3, 18 patients will be allocated to group 4

Clinical data on patients will be recorded in forms in the Clinical Research Facility.

2.4 Trial Flowchart (weeks 1-8)

WEEKS		W1							W2							W3							W4							W5							W6							W7							W8						
DAY	PR	PT																																																							
Group 1: Watch & Wait																																																									
Physical exam. / safety check	+	+																					+																				+													
Blood test (40ml)		+																					+																				+													
CT scan	+																																																								
Group2: Cyclophosphamide																																																									
Physical exam. / safety check	+	+																					+							+														+													
Blood test (40ml)		+							+							+							+							+														+													
CT scan	+																																																								
Blood for FACS (10ml)					+														+																																					
Cyclophosphamide		≡	≡	≡	≡	≡	≡	≡								≡	≡	≡	≡	≡	≡																																				
Phone to check safety/compliance			░			░											░			░																																				
Group 3: TroVax®																																																									
Physical exam. / safety check	+	+																					+							+														+													
Blood test (40ml)		+																					+							+														+													
CT scan	+																																																								
TroVax® injection																							▧														▧														▧						
Group4: Cyclophosphamide + TroVax®																																																									
Physical exam. / safety check	+	+																					+							+														+													
Blood test (40ml)		+							+							+							+							+														+													
CT scan	+																																																								
Blood for FACS (10ml)					+														+																																					
Cyclophosphamide		≡	≡	≡	≡	≡	≡	≡								≡	≡	≡	≡	≡	≡																																				
Phone to check safety/compliance			░			░											░			░																																				
TroVax® injection																							▧														▧														▧						

PR: Pre-recruitment

PT: Pre-treatment

Bloods are taken BEFORE dosing

≡ Cyclophosphamide 50mg ▧ TroVax® i.m 1ml injection ░ Phone check

Trial Flowchart cont. (weeks 9-16)

WEEKS	W9							W10							W11							W12							W13							W14							W15							W16						
DAY																																																								
Group 1: Watch & Wait																																																								
Physical exam. / safety check																						+																												+						
Blood test (40ml)																						+																												+						
CT scan																						+																																		
Group 2: Cyclophosphamide																																																								
Physical exam. / safety check								+														+																												+						
Blood test (40ml)								+														+																												+						
CT scan																						+																																		
Group 3: TroVax®																																																								
Physical exam. / safety check								+														+																												+						
Blood test (40ml)								+														+																												+						
CT scan																						+																																		
TroVax® injection																																																								
Group 4: Cyclophosphamide + TroVax®																																																								
Physical exam. / safety check								+														+																												+						
Blood test (40ml)								+														+																												+						
CT scan																						+																																		
TroVax® injection																																																								

TroVax® i.m 1ml injection

Bloods are taken BEFORE dosing

3 TRIAL MEDICATION

3.1 Investigational Medicinal Product

Background information about the format, stability and safety profile of the IMPs used in TaCTiCC is given in Appendix 1.

Cyclophosphamide has been widely used for the treatment of different solid tumours including CRC. In this study we are using much lower doses of cyclophosphamide which has previously been shown to have minimum side effects [16, 17]. Cyclophosphamide tablets of 50mg (Pharmacia Ltd brand) will be purchased by the host institution for use in the trial and stored in the pharmacy clinical trials department. Cyclophosphamide used in this trial will be dispensed from the pharmacy following receipt of a trial-specific prescription and labelled according to the Medicine for Human Use (Clinical Trials) Regulation 2004. Trial subjects will be provided with the exact quantity of cyclophosphamide needed for each treatment course.

5T4 is an oncofetal antigen expressed in a wide spectrum of cancers but not in most normal tissues, making it an attractive vaccine candidate. It has been expressed in the non-replicating pox virus vector modified vaccinia ankara (MVA) (produced by Oxford BioMedica, trade name TroVax®). TroVax® is an unlicensed medicinal product that is manufactured, labelled and released by a qualified person in compliance with the Medicine for Human Use (Clinical Trials) Regulation 2004 and Good Manufacturing Practice by Oxford Biomedica before being supplied to the host institution. TroVax® is supplied as a lyophilised powder that is stored at 2 to 8°C. TroVax® will be stored in the pharmacy clinical trials refrigerator and daily temperature monitoring will be performed. TroVax® will be dispensed from the pharmacy following receipt of a trial specific prescription. More details about the handling, storage, reconstitution and disposal are given in Appendix 2.

3.2 Dosing Regimen

TroVax® will be administered by trial nurses with cover by one of the trial clinicians, whose contact details are shown on page 2. Cyclophosphamide is an oral tablet self-administered by the patient.

Group 1: Watch and wait, i.e. no additional treatment unless clinically indicated
Group 2: Metronomic cyclophosphamide 50mg bd as single agent on week 1 (14 doses) and on week 3 (12 doses to make it comparable to group 4).
Group 3: Vaccination (i.m.) TroVax® (1×10^9 $TCID_{50}$) at week 4, 6, 8, 10, 12 and 16.
Group 4: Metronomic cyclophosphamide 50 mg bd on weeks 1 (14 doses) and week 3 (12 doses instead of 14 to allow for extra 24 hour wash-out time), followed by TroVax® (1×10^9 $TCID_{50}$) on weeks 4, 6, 8, 10, 12 and 16.

TroVax® is an i.m. injection administered by clinical staff in the Clinical Research Facility. 1×10^9 $TCID_{50}$ in 1 ml will be administered and the patient observed for 60 minutes post injection. The doses will be given every 2 weeks for the first 5 injections, with an additional dose 4 weeks after. If patients receiving Trovax are within the ranges defined in section 7.3, and are in a stable condition, further TroVax® injections will be offered every 8 weeks.

Under these conditions, patients may be required to undergo further medical assessments and provide additional samples in order to monitor ongoing Treg depletion. The possibility of further investigations under these circumstances is specifically described in the patient information sheet.

Patients who responded to cyclophosphamide will be offered a further course/s at a dose and frequency as previously given. Patients who do receive further trial medication will be medically assessed as part of their on-going care, and will be asked to provide additional samples to monitor the effects, as described in the patient information sheet.

3.3 Drug accountability

The pharmacy department at UHW will develop trial specific prescriptions and accountability logs for the TroVax® and cyclophosphamide and maintain full drug accountability during the trial.

3.4 Subject compliance

Group 1: Watch and wait. No compliance measures are necessary.

Group 2: Metronomic cyclophosphamide. Patients will be contacted by phone during their treatment course to remind them to take the cyclophosphamide tablets. Patients will also be reminded to bring back cyclophosphamide containers to their clinic appointment. Empty/unused tablets will be returned to pharmacy and unused tablets will be counted by pharmacy clinical trials staff and recorded in the accountability log.

Group 3: Vaccination (i.m.) TroVax®. No compliance measures are necessary.

Group 4: Metronomic cyclophosphamide followed by TroVax®. Patients will be contacted by phone during their treatment course to remind them to take the cyclophosphamide tablets. Patients will also be reminded to bring back cyclophosphamide containers to their clinic appointment. Empty/unused tablets will be returned to pharmacy and unused tablets will be counted by pharmacy clinical trials staff and recorded in the accountability log.

3.5 Concomitant medication

Patients should not be receiving cancer chemotherapy at the time of randomisation and have stable disease (see inclusion / exclusion criteria on sections 4.1 and 4.2).

Palliative medication may be given as required by the clinician. Relevant concomitant medications will be recorded.

4 SELECTION & WITHDRAWAL OF TRIAL SUBJECTS

4.1 Inclusion criteria

- Patient able to give informed consent personally
- Signed and dated written informed consent
- Age ≥ 18 years
- Clinical diagnosis of inoperable colorectal cancer
- WHO performance status 0-2 (see Appendix 3 for WHO scale information)
- Responding or stable disease as defined by oncologist following 12 weeks of front-line chemotherapy for metastatic disease, as demonstrated on CT scan in comparison with pre-treatment CT scan (RECIST), within 4 weeks of trial entry
- Any cancer related symptoms are under control with standard non-chemotherapy medications
- Subject has adequate bone marrow function as defined by an Absolute Lymphocyte Count ≥ 500/µL, Absolute Neutrophil Count >1200/µL, Platelet Count >100,000/µL

4.2 Exclusion criteria

- Patient unable to give informed consent personally
- Creatinine level >1.5 Upper Limit of Normal (ULN)
- Bilirubin level >50 µmol/l
- Alkaline Phosphatase >3 ULN
- Aspartate Aminotransferase and Alanine Aminotransferase >2 ULN
- Prothrombin time >18sec
- Prior exposure to TroVax®
- Life expectancy of less than 3 months

- Patient has relapsed
- Diagnosed as being immunosupressed, receiving oral steroids (> prednisolone 10 mg daily) (nasal sprays and inhalers are permitted) or receiving immunosuppressive therapy for oncology disorders, or following transplant
- Patient has completed chemotherapy less than 2 weeks from the start of the treatment
- Subject has clinically apparent/active autoimmune disease (prior confirmed diagnosis or treatment for autoimmune disease including Systemic Lupus Erythematosis, Grave's disease, active Hashimoto's thyroiditis, multiple sclerosis, insulin dependent diabetes mellitus and rheumatoid arthritis). Note: subjects with non-insulin dependent diabetes mellitus can be included, as can subjects with controlled and rarely flaring rheumatoid disease and end-stage insulin dependent diabetes mellitus controlled on insulin.
- Subject has a platelet count prior to start of chemotherapy >400,000/µL; Monocytes >80,000/ µL; Haemoglobin <11 g/dL
- Significant cancer related symptoms requiring immediate treatment with chemotherapy
- "Currently active" second malignancy, other than non-melanoma skin cancer and previously diagnosed prostate cancer or breast cancer which is stable clinically for more than 5 years with or without hormone treatment. Subjects are not considered to have a "currently active" malignancy if they have completed therapy more than 5 years previously and have no known evidence of residual or recurrent disease
- Evidence of significant clinical disorder or laboratory finding which in the opinion of the investigating physician makes it undesirable for the patient to participate in the trial. No participant should have a serious or uncontrolled intercurrent infection (including those positive for HIV)

- Psychiatric illnesses/social situations that limit compliance with protocol requirements
- Allergy to egg proteins, cyclophosphamide, neomycin or allergic response to vaccinia vaccines
- Known cerebral metastases (known from previous investigations or clinically detectable)
- Haemorrhagic cystitis
- Severe infection
- They are pregnant or lactating.
- Individuals with the potential of child bearing age and unwilling to take two forms of contraception

4.3 Selection of participants

At the University Hospital of Wales at any one time, approximately 50 patients are being treated with palliative chemotherapy for advanced disease. Median survival of these patients is 16-20 months. In the MRC COIN trial the median disease progression free survival following completion chemotherapy in the intermittent (arm C) cohort was 4.6 months [18].

Subjects with palliative disease undergoing active treatment are reviewed regularly in outpatients at the clinical investigators' hospital. Suitable patients will be approached in clinic and if willing to consider participation, given information sheets.

4.4 Randomisation procedure

Patients will present to one of the collaborators in their clinic, as a part of the patient's routine clinical assessments. If appropriate, i.e. stable disease after approximately 12 weeks, patients will undergo a break from chemotherapy ("chemo holiday"), based on the results of a CT scan and clinical assessment.

The trial will be introduced to the patient at this point, at the beginning of their chemo holiday. Patients will be given sufficient time to consider the trial (at least 24 hours), before providing written consent. The right to withdraw consent at any time will be re-iterated to the patient.

The Trials Office will undertake the randomisation using an un-stratified balanced block design, and communicate the outcome to the collaborator immediately upon randomisation.

4.5 Withdrawal of subjects

In accordance with the regulations in force, subjects are free to withdraw at any time, without giving a reason, and without their medical care or legal rights being affected.

Additionally, patients will be withdrawn from treatment if the CRC disease has progressed (as defined either by evidence of radiological progression on the scheduled CT scans or because of clinical deterioration as assessed by the oncologist) or there appears to be toxicity associated with the trial medication (please refer to section 7.3 for more details)

Withdrawn patients will continue their standard follow up care under the oncologists and their clinical details will be recorded at each clinic visit. They will be followed-up for pharmacovigilance purposes for 6 months after withdrawal.

4.6 Expected duration of trial

It is suggested that enrolling 54 patients over a 24-30 month period, with a 12 month follow up period after the final patient entry is a realistic proposition based on current patient numbers.

5 TRIAL PROCEDURES

5.1 By visit

Please refer to the table shown in section 2.4 for details of the visit schedule.

In addition to their scheduled visits the patients will be seen regularly by their clinical oncology team.

Patients will be commenced on therapy within 4 weeks of their baseline scan where possible..

If the patient is well enough to attend the Clinical Research Facility, there will be no exclusions to obtaining a blood sample as long as the treating clinician deems the individual well enough to give the sample. All blood samples will be taken before any treatment is given.

5.2 Sample collection

Group 1: 40 ml blood weeks 1, 4, 7 11, 15
Group 2: 40 ml blood weeks 1, 2, 3, 4, 5, 7, 9, 11, 13, 15 (additional 10 ml blood for FACS week 1 and 3)
Group 3: 40 ml blood weeks 1, 4, 5, 7, 9, 11, 13, 15.
Group 4: 40 ml blood weeks 1, 2, 3, 4, 5, 7, 9, 11, 13, 15 (additional 10 ml blood for FACS week 1 and 3)

CT scan of abdomen / chest will be performed as part of the standard clinical care at week 12.

Patients may continue in the clinical trial as long as there is no disease progression as determined by the clinician.

5.3 Laboratory tests

Full clinical assessment and routine blood tests (including autoantibodies, thyroid function tests and blood glucose at baseline) will be conducted during prior outpatient appointments to ensure eligibility.

Tumour burden will be assessed quantitatively using RECIST criteria at 12-weekly intervals as per standard practice. On clinical evidence of progressive disease, the patients would be treated with standard chemotherapy as indicated.

6 ASSESSMENT OF EFFICACY

6.1 Primary efficacy parameters

I. Development or increase in anti-5T4 i) T cell responses ii) B cell responses (antibodies) in patients treated with cyclophosphamide and TroVax® versus TroVax® alone or cyclophosphamide alone or untreated patients **at week 7**

6.2 Secondary efficacy parameters

I. Development or increase in anti-5T4 i) T cell responses ii) B cell responses (antibodies) in patients treated with cyclophosphamide and TroVax® versus TroVax® alone or cyclophosphamide alone or untreated patients

II. Overall Survival as the time in days from randomisation until death of any cause censoring at date of last follow-up

III. Time To Progression with death as a competing risk will be measured as the time in days from randomisation until disease progression as determined by RECIST criteria for radiological imaging and clinical assessment

IV. Progression Free Survival will be measured as the time in days from randomisation until progression or death of any cause censoring at date of last follow up

V. Incidence, nature, severity, relatedness and seriousness of treatment-emergent adverse events and clinically significant abnormal laboratory results

VI. Change in the frequency of Tregs measured in blood samples in patients treated with metronomic cyclophosphamide compared to patients not receiving cyclophosphamide at week 4

6.3 Procedures for assessing / measuring efficacy parameters

From 40 ml blood sample it is expected to obtain at least 25 million PBMCs. Extraneous peripheral blood mononuclear cells will be frozen for any additional later analysis. Patients will be consented for storage of samples and subsequent research.

6.3.1 Primary efficacy parameters

I. Measurement of T cell responses **at week 7 time point**. i) IFNγ ELISPOT assay will be used to measure *ex vivo* and cultured T cell responses to tumour antigens and control antigens (TT and PPD proteins). Ex vivo responses will be measured in the presence or absence of $CD4^{+}CD25^{hi}$ cells, using methods of depletion devised in our laboratory. Detailed analysis of cognate $CD4^{+}$ / $CD8^{+}$ T cell function will also be performed using the Aria II flow cytometer to simultaneously analyse intracellular production of IL-2, IFNγ, TNFα and IL-10. 6 day proliferation assays using ^{3}H-thymidine incorporation will be carried out using the same cell preparations. To perform these assays will require approximately 20 million peripheral blood mononuclear cells (PBMCs).

II. Measurement of anti-5T4 and anti-MVA serum antibody titres **at week 7**

time point by standard ELISAs (using serum from preparation of above PBMC sample)

6.3.2 *Secondary efficacy parameters*

I. Kinetics of T and B cell responses will be measured using the methods detailed in point 6.3.1 at the different time points detailed in section 5.2

II. Overall Survival will be obtained subtracting the date of randomisation to the date of death to obtain the number of days the patient survived after randomisation

III. Time To Progression with death as a competing risk will be obtained subtracting the date of randomisation to the date of disease progression to obtain the number of days elapsed from randomisation until progression of disease

IV. Progression Free Survival will be obtained subtracting the randomisation date to the date of disease progression or death of any cause

V. Toxicity will be measured using the NCI CTC (National Cancer Institute Common Toxicity Criteria) v3 scale; toxicity grading will be recorded in the patient's CRF.

VI. T cell phenotyping and enumeration will be performed by flow cytometry. T cells will be stained for a variety of markers to assess the effect of treatment on the phenotype and frequency of different T cell subsets including Tregs. Markers will include CD4, CD8, CD25, CTLA4, CD45RA, CD45RO, GITR and Foxp3. Requires maximum of 1 m cells, and will be combined with the flow cytometry analysis outlined above.

7 ASSESSMENT OF SAFETY

7.1 Specification, timing and recording of safety parameters

At regular intervals (specified in section 2.4) the subjects will be screened for the following safety parameters:

- General physical examination (vital signs / heart / lungs / abdomen)
- Full blood count
- Urea and electrolytes
- Liver function tests

The above will be recorded in the patients' case report form (CRF) by the relevant clinician or their nominee.

TroVax® has already been safely combined with chemotherapy in patients with colorectal cancer [20]. In this study it will be given sequentially after low dose cyclophosphamide. As this sequential usage is novel, it will therefore be subject to enhanced safety monitoring. Reports of recorded events, with particular emphasis on unexpected grade IV toxicities, along with a narrative from Chief Investigator, will be submitted to the Data Monitoring and Ethics Committee (DMEC) after every 6 completed patients in Group IV. Safety in this group will be assessed weekly for a minimum of the first 4 weeks, following established procedures within the HCTU group.

7.2 Adverse event reporting

7.2.1 Definitions

The Medicines for Human Use (Clinical Trials) Regulations 2004 and Amended Regulations 2006 provides the following definitions:

Adverse Event (AE)

Any untoward medical occurrence in a subject to whom a medicinal product has been administered including occurrences which are not necessarily caused by or related to that product.

Adverse Reaction (AR)

Any untoward and unintended response, in a subject, to an investigational medicinal product, that is related to any dose administered to that subject.

Unexpected Adverse Reaction (UAR)

An adverse reaction the nature and severity of which is not consistent with the information about the medicinal product in question set out in:

- the summary of product characteristics (SmPC) for that product (for products with a marketing authorisation, i.e. cyclophosphamide).
- the Investigator's Brochure (IB) relating to the trial in question (for any other investigational product, i.e. TroVax®).

Serious Adverse Event (SAE)

Any adverse event, adverse reaction or unexpected adverse reaction, respectively, that:

- results in death
- is life-threatening
- required hospitalisation or prolongation of existing hospitalisation
- results in persistent or significant disability or incapacity
- consists of a congenital anomaly or birth defect

Suspected Unexpected Serious Adverse Reaction (SUSAR)

Any adverse reaction that is classed in nature as serious and which is not consistent with the information about the medicinal product in question set out

- in the case of a licensed product, the summary of product characteristics (SmPC) for that product (i.e. cyclophosphamide)
- In the case of any other investigational medicinal product, the Investigator's Brochure (IB) relating to the trial in question (i.e. TroVax®)

Important medical events that may not be immediately life-threatening or result in death or hospitalisation but may jeopardise the patient or may require intervention to prevent one of the other outcomes listed in the definition above should also be considered serious.

The patient population being studied in this trial is palliative and it is expected that >50% will die as a result of their tumour during the study. Death or hospitalisation as a result of disease progression and other events that are primary or secondary outcome measures are not considered to be SAEs and should be recorded in the normal way, in the subjects' CRFs.

More specifically, the following events **do not** require reporting as SAEs:

- Hospitalisation or prolongation of hospitalisation <u>as a result of progression of disease</u>
- Death as a result of progression of disease

7.2.2 Reporting responsibilities

Cardiff University as sponsor have delegated the Sponsor's responsibility for Pharmacovigilance (as defined in Regulation 5 of the Medicines for Human Use (Clinical Trials) Regulations 2004) to the HCTU Trials Office.

Investigators have an obligation to report any SAE (excepting those specified in this protocol as not requiring reporting in section 7.2.1) that occurs in a subject, within 24 hours, to the Trials Office. Investigators will be asked to record their opinion as to

whether the SAE, as defined above, was related to the study medication. This will also subject to review by the DMEC.

Within the TaCTiCC trial, any event that occurs within 30 days of the patient's treatment and meets the criteria laid out in section 7.2.1 of the protocol should be reported as an SAE. Beyond this period, any event that is causally linked to treatment received as part of the trial, and meets the criteria laid out in the protocol, should also be reported. The immediate report to the Trials Office must be made in writing using the SAE Report form provided and, if required, the Trials Office will request further details. Where the event reported consists of, or results in, the death of a subject, the Sponsor may request further details from the Investigator. Where the death has been reported to the relevant ethics committee, the committee may request further details from the Investigator.

The Chief Investigator (CI) or their nominee will review and record all SAE reports received by the Trials Office. The CI will be responsible for reporting the events, when required, to the MHRA, Ethics and DMEC according to the appropriate timelines.

Suspected unexpected serious adverse reactions (SUSARs) which occur during the course of the clinical trial will be reported within 7 days of the Trials Office becoming aware of the event, to the MHRA and the relevant ethics committee. Any additional relevant information will be sent within eight days of the initial report being sent. All SUSAR reports or information will be provided to the MHRA using the eSUSAR reporting system. The relevant research ethics committee will be informed of any SUSARs using the CIOMS form.

In addition to the expedited reporting required for SUSARs, the Trials Office will periodically submit a safety report to both the MHRA and the relevant Ethics Committee. This will be annually from the date of the CTA approval. The annual

safety report will take into account all new available safety information received during the reporting period.

An independent DMEC will be convened to monitor safety and scientific integrity of the clinical study, to assess risk versus benefit, and to recommend any changes warranted to the study design. The independent DMEC will meet to review study data from the clinical database, including safety and primary response data. DMEC decisions will be recorded as minutes in the Trial Master File. For details about meetings and composition, please refer to section 9.

7.3 Treatment stopping rules

Treatment will be stopped if:

- Clinical relapse / progression of tumour necessitating a recommencing of chemotherapy as assessed by clinicians
- If full blood count falls in indices of >30% then stop experimental therapy with cyclophosphamide / vaccine
- If electrolytes and liver function tests increase > 1.5 ULN then stop active treatment with cyclophosphamide / vaccine

8 STATISTICS

The trial aims to recruit a total of 54 patients to a 2x2 factorial design. Patients will be randomised in a 1:1 fashion between receiving cyclophosphamide and not, and in a 2:1 fashion between receiving TroVax® and not. This gives rise to four treatment arms:

- watch and wait (9 patients)
- cyclophosphamide alone (9 patients)
- TroVax® alone (18 patients)
- TroVax® plus cyclophosphamide (18 patients)

Comparisons of TroVax® versus not and cyclophosphamide versus not will be performed stratified for the other treatment allocation, and interactions between the treatments will be specifically tested for.

Consequently 5 out of every 6 patients will receive some experimental therapy. Overall 36 patients will receive TroVax® versus 18 receiving none, and 27 will receive cyclophosphamide versus 27 receiving none.

Both the cyclophosphamide and TroVax® randomisation will recruit 18 patients to each arm. Based on the primary endpoints, this gives greater than 80% power to detect a difference of one standard deviation[3] in measured T/B cell responses at $p<0.05$. Alternatively there will be 80% power to detect a 50% absolute difference in the proportion of responders (e.g. from 25% to 75%).

8.1 Sample size

Two comparisons will be carried out, and power is based on the average effect size expected for each treatment (allowing for the possible synergy between the two treatments). All tests will be carried out at the 5% significance level. A randomisation of 27 vs 27 patients to receive cyclophosphamide or not gives 80% power to detect a moderate to large difference of 0.8 s.d. in antitumour response, or other laboratory markers. For the TroVax® randomisation, a total of 54 patients (allocated as 36 vs 18) gives 80% power to detect a difference of 0.83 points between TroVax® and placebo. This would be equivalent to a difference of 1 sd for TroVax® + cyclophosphamide vs cyclophosphamide and a smaller difference of 1/3 s.d. for TroVax® alone versus watch and wait, or 1.25 s.d. in the cyclophosphamide group and no difference in the no cyclophosphamide group. As the possible synergy between cyclophosphamide and TroVax® is of interest, the TroVax® randomisation is

[3] In a cohort of > 50 patients studied pre-operatively the mean 5T4 T cell response before / after in vitro Treg depletion is 7.15 (SD 1.7) vs. 16.0 (SD 2.9) T cells / million PBMCs. We expect the responses generated by vaccination to be far more robust in the light of previous phase I/II studies giving TroVax® to CRC patients. An increase of at least 1 SD is expected if Treg depletion in vivo is successful. Frequency of Tregs in this population is approximately 1-3 % of CD4+ T cells in PBMCs and we have previously measured a significant increase in patients vs. age matched controls [11].

in a ratio of 2:1; if such synergy is seen, then a comparison of adding cyclophosphamide to TroVax® will contain 36 patients, enough to see a 1 s.d. difference with 80% power.

8.2 Randomisation

The trial will be open label; given the objective nature of the measurements, this is unlikely to introduce bias. Randomisation will be by means of a fax and a telephone call to the trials office. Allocation will be performed using a balanced-block randomisation without stratification.

8.3 Analysis

Continuous variables will be analysed using standard t-tests, regression approaches (for stratified and multivariable analyses) or Wilcoxon rank-sum tests as appropriate. Proportions will be compared using the Mantel-Haenszel test. Where possible, effect sizes and confidence intervals will be given. The primary analysis of treatment effect for each treatment will be performed stratified for the other treatment allocated, and a test for interaction performed. However, because of the well-known problems with subgroup analyses, and given the small numbers, any analyses based on other baseline covariates will be purely exploratory and hypothesis-generating, and suitable standard tests for heterogeneity and trend will be used throughout.

Analyses will be by intention to treat – there will be no imputation performed for missing data.

9 DATA MONITORING & ETHICS COMMITTEE

Data will be provided to the Data Monitoring and Ethics Committee (DMEC) by the Chief Investigator and the study's statistician. The DMEC will examine the accumulating safety and efficacy data from the trial. Their remit may include recommending trial closure on safety grounds. Because the outcome measures

chosen here are surrogates, and given the small size of the trial, there are no formal provisions to stop the trial early because of efficacy. After each meeting they will write a letter giving recommendations to the Sponsor.

The DMEC will be composed of the following members:

- Dr John Staffurth, Institute of Cancer and Genetics, School of Medicine, Cardiff University
- Prof Alan Burnett, Department of Haematology, Cardiff University
- Prof Tim Elliot, Chair of Experimental Oncology and Associate Dean for the Faculty of Medicine, University of Southampton

10 DIRECT ACCESS TO SOURCE DATA AND DOCUMENTS

The Investigator will permit trial-related monitoring, audits, GTAC review, and regulatory inspections (where appropriate) by providing direct access to source data and other documents (i.e. patients' case sheets, blood test reports, CT-scan reports, histology reports etc). Patients will provide specific consent to grant access to these data.

11 ETHICS & REGULATORY APPROVALS

The trial will be conducted in compliance with the principles of the Declaration of Helsinki (1996), the principles of GCP and in accordance with all applicable regulatory requirements, including but not limited to, the Research Governance Framework and the Medicines for Human Use (Clinical Trial) Regulations 2004, as amended in 2006 and any subsequent amendments.

This protocol and related documents will be submitted for review to the Sponsor and the Gene Therapy Advisory Committee (GTAC) for ethical approval, and to the

Medicines and Healthcare products Regulatory Agency (MHRA) for Clinical Trial Authorisation.

Annual progress and safety reports and a final report at conclusion of the trial will be submitted to GTAC and the MHRA within the timelines defined in the Regulations.

12 QUALITY ASSURANCE

The Trials Office has experience in the conduct and management of clinical trials across the phases, and will adhere to standard operating procedures (SOPs), both generic and study-specific, during the conduct of this trial.

Collection, retention and entry of source data at the Clinical Research Facility on the Heath Park Campus will also be carried out to developed clinical trials practices.

An element of source data verification (SDV) may be required during the trial, in order to present data to the DMEC. The procedure for SDV will be outlined in a trial-specific SOP, with reference to the requirements of the Sponsor.

13 DATA HANDLING

The Chief Investigator will act as custodian for the trial data. Data will be retained and handled with strict adherence to Cardiff University Research Governance Framework. More specifically:

- patient data will be anonymised
- all anonymised data will be stored on a password protected computer
- all trial data will be stored and archived in line with the Medicines for Human Use (Clinical Trials) Amended Regulations 2006

Archiving will be done in accordance with Cardiff University archiving policy.

14 PUBLICATION POLICY

It is intended that the results of the study will be reported and disseminated at international conferences and in peer-reviewed scientific journals. The patients will be consented to publish their anonymised data only, and will be informed when a publication is imminent.

15 FINANCIAL ASPECTS AND INSURANCE

Oxford Biomedica will provide investigational medicinal product (TroVax®) and funding for laboratory aspects, in support of the trial.

Insurance for aspects of the trial are provided by the Sponsor’s clinical trial indemnity.

16 REFERENCES

1. Dunn, G.P., et al., Cancer immunoediting: from immunosurveillance to tumor escape.[see comment]. *Nature Immunology*, 2002. 3(11): p. 991-8.

2. Penn, I., Posttransplant malignancies. *Transplantation Proceedings* 1999. 31(1-2): p. 1260-2.

3. Godkin, A. and A. Gallimore, CD4+CD25+ regulatory T cells and tumour immunity-observations in mice and men*. Immunology* 2008. 123(2): p. 157-63.

4. Galon, J., at al., Type, density and location of immune cells within human colorectal tumors predict clinical outcome. Science 2006. 313(5795): p. 1868-9.

5. Dudley, M.E., et al., Cancer regression and autoimmunity in patients after clonal repopulation with antitumor lymphocytes. *Science* 2002, 298(5594): p. 850-4.

6. Hunder, N.N., et al., Treatment of metastatic melanoma with autologous CD4+ T cells against NY-ESO-1*. N Engl J Med* 2008. 358(25): p. 2698-703.

7. Sakaguchi, S., Naturally arising Foxp3-expressing CD25+CD4+ regulatory T cells in immunological tolerance to self and non-self. *Nature Immunology* 2005. 6(4): p. 345-52.

8. Jones, E., et al., Depletion of CD25+ regulatory cells results in suppression of melanoma growth and induction of autoreactivity in mice. *Cancer Immunity* 2002. 2(1): p. 22.

9. Betts, G., et al., The impact of regulatory T cells on carcinogen-induced sarcogenesis. *Br J Cancer* 2007. 96(12): p. 1849-54.

10. Curiel, T.J., et al., Specific recruitment of regulatory T cells in ovarian carcinoma fosters immune privilege and predicts reduced survival.[see comment]. *Nature Medicine* 2004. 10(9): p. 942-9.

11. Clarke, S.L., et al., CD4+CD25+FOXP3+ regulatory T cells suppress anti-tumor immune responses in patients with colorectal cancer. *PLoS ONE* 2006. 1: p. e129.

12. Mackett, M., et al., Vaccinia virus: a selectable eukaryotic cloning and expression vector. *Proc. Natl. Acad. Sci. USA*. 1982. 79: 7415-7419.

13. Amato R.J. 5T4-modified vaccinia Ankara: progress in tumor-associated antigen-based immunotherapy. *Expert Opin Biol Ther*. 2010. Feb;10(2):281-7. Review.

14 Harrop, et al., Cross-trial analysis of immunologic and clinical data resulting from PhaseI and II trials of MVA-5T4 (TroVax) in colorectal, renal and prostate cancer patients. *J Immunother*. 2010. 33(9): 999-1005.

15 Amato R. J., et al., Vaccination of metastatic renal cancer patients with MVA-5T4: a randomized, double-blind, placebo-controlled Phase III study. *Clin Cancer Res*. 2010. 16(22): 5539-47.

16. Ghiringhelli, et al., Metronomic cyclophosphamide regimen selectively depletes CD4+CD25+ regulatory T cells and restores T and NK effector functions in end stage cancer patients. *Immunotherapy* 2007, 56(5):641-8.

17 Viaud et al., Cyclophosphamide induces differentiation of Th17 cells in cancer patients. *Cancer Res.* 2010. 71(3): 661-5

18. Maughan et al., Comparison of intermittent and continuous palliative chemotherapy for advanced colorectal cancer: a multicentre randomised trial.*Lancet* 2003, 361(9356):457-64.

19. Adams R, Wilson R, Seymour M, Meade AM, Madi A, Cassidy J, Fisher D, Kenny S, Kaplan R, Maughan TS on behalf of the COIN Trial Investigators. Intermittent vs continuous oxaliplatin-fluoropyrimidine (Ox-Fp) chemotherapy (CT) in first-line treatment of patients (pts) with advanced colorectal cancer (aCRC): predictive factors (PF), Quality of Life (QL) and final efficacy results from the MRC COIN trial. ASCO Annual Meeting 2010, Abstract Number 53047

20 Harrop et al. Vaccination of colorectal cancer patients with TroVax given alongside chemotherapy (5-fluorouracil, leukovorin and irinotecan) is safe and induces potent immune responses. *Cancer Immunol Immunother*. 2008; 57(7): 977-86

17 SIGNATORIES

Chief Investigator: Dr Andy Godkin

Signature: ______________________ Date: ___________________

Statistician: Dr Robert Hills

Signature: _______ Date: ___________________

APPENDIX 1 Preparation, Administration and Handling of Investigational Medicinal Products

Cyclophosphamide Tablets 50 mg (Pharmacia Limited)

Cyclophosphamide is supplied as white polyethylene containers with polyethylene snap-caps, containing a white capsule of desiccant. Cyclophosphamide is an alkylating, antineoplastic agent. Cyclophosphamide has been used successfully to induce and maintain regressions in a wide range of neoplastic conditions, including leukaemias, lymphomas, soft tissue and osteogenic sarcomas, paediatric malignancies and adult solid tumours; in particular, breast and lung carcinomas.

Posology and method of administration

Route of administration: Oral.

In this study we are using a metronomic dose of 50mg bd.

Cyclophosphamide tablets should be swallowed whole, preferably on an empty stomach, but if gastric irritation is severe, they may be taken with meals. With the very low dose of drug used in this study, side effects (i.e. urinary tract etc) are unlikely, but the patient should be well hydrated and maintained in fluid balance.

The patient should be well hydrated and maintained in fluid balance.

Mesna (Uromitexan) can be used concurrently with cyclophosphamide to reduce urotoxic effects (for dosage see Uromitexan data sheet). If Mesna (Uromitexan) is used to reduce uroethelial toxicity, frequent emptying of the bladder should be avoided.

Due to the reduced dose of this study, we do not expect that the patients develop haematological toxicity. However, may the leucocyte count be below 4,000/mm3 or the platelet count below 100,000/mm^3, treatment with cyclophosphamide should be temporarily withheld until the blood count returns to normal levels.

Contraindications

Cyclophosphamide is contraindicated in patients with hypersensitivity and haemorrhagic cystitis.

Special warnings and precautions for use

Cyclophosphamide should be withheld in the presence of severe bone marrow depression. Regular blood counts should be performed in patients receiving cyclophosphamide.
It should not normally be given to patients with severe infections and should be withdrawn if such infections become life threatening.

Cyclophosphamide should be used with caution in debilitated patients and those with renal and/or hepatic failure.

Cyclophosphamide should be used only under the directions of physicians experienced in cytotoxic or immunosuppressant therapy.

This product should not normally be administered to patients who are pregnant or to mothers who are breast feeding. Alkylating agents, including cyclophosphamide, have been shown to possess mutagenic, teratogenic and carcinogenic potential. Pregnancy should therefore be avoided by appropriate means during cyclophosphamide therapy and for three months thereafter.

Although it is less likely with the patient demography in this study, if appropriate males receiving cyclophophamide will have the hazards explained, and the necessity of contraception upto 3 months after ceasing treatment.

Overdose

Myelosuppression (particularly granulocytopenia) and haemorrhagic cystitis are the most serious consequences of overdosage. Recovery from myelosuppression will occur by the 21st day after the overdosage in the great majority of patients (at doses up to 200 mg/kg i.v.) while granulocytopenia is usually seen by day 6 and lasts for a mean period of 12 days (up to 18 days). A broad spectrum antibiotic may be administered until recovery occurs. Transfusion of whole-blood, platelets or white cells and reverse barrier nursing may be necessary.

If the drug has been taken in the form of tablets, early gastric lavage may reduce the amount of drug absorbed.

During the first 24 hours and possibly up to 48 hours after overdosage, i.v. Mensa may be beneficial in ameliorating damage to the urinary system. Normal supportive measures such as analgesics and maintenance of fluid balance should be instituted. If the cystitis does not resolve more intensive treatment may be necessary.

No further courses should be given until the patient has fully recovered.

Storage and Shelf life

36 months. Do not store above 25°C. Store in the original container in order to protect from moisture.

TroVax® (Oxford BioMedica)

The TroVax® active substance is a highly attenuated recombinant vaccinia virus that contains a gene encoding the human 5T4 oncofetal antigen. The vector is derived from a replication-incompetent vaccinia virus called "MVA" that was developed and used as a safe vaccine for smallpox. MVA cannot replicate to produce infectious virus in any primary human cell type. TroVax® is formulated as a lyophilized powder and reconstituted in sterile water for injection prior to administration. It is administered as an i.m. injection (for more information on handling and administration see App. 2).

Undesirable Effects

The only adverse reactions consistently attributed to TroVax® are self limiting febrile reactions and mild injection site reactions.

Contraindications

TroVax® is currently contraindicated in women of child-bearing potential and in patients who are allergic to egg protein and/or exhibit sensitivity to neomycin and/or have active eczema with lesion(s) on the skin. It is also contraindicated in patients who are clinically immunosuppressed or who take corticosteroids or other immunosuppressant agents.

Pregnant or lactating patients must not receive TroVax®. Likewise, personnel who are pregnant should not handle TroVax® or waste contaminated with TroVax®. Personnel of child bearing potential should be informed of the potential risk. Personnel who are immunologically compromised should also not handle TroVax® or waste contaminated with TroVax®.

In addition, since the placenta expresses 5T4, TroVax® could potentially induce antibodies that might harm fetal growth and development. These phenomena have not been observed, however women of child bearing potential should only be given TroVax® if the clinical benefit:risk assessment is favourable. Two reliable methods of contraception should be used during treatment with TroVax® and continued for an appropriate length of time afterwards; one month for male patients and three months for female patients. If pregnancy should occur, treatment with TroVax®

should be suspended.

Overdosage

TroVax® has been given to animals in multiples of up to 280 times the clinical dose, based on pfu/kg, without toxic effects. Overdosage has not been documented in humans.

Storage and Shelf Life

TroVax® is presented as a sterile powder in 2 ml glass vials with 13 mm rubber stopper and combi caps. Specifications for the container and closure system are in compliance with International Conference on Harmonization (ICH) guidelines. The container and closure have been validated to demonstrate the integrity of closure and its effectiveness as a microbial barrier. The bottles and stoppers are suitable for the proposed storage conditions at ≤-15°C for 12 months then at 2-8°C for 12 months. Once TroVax® is re-constituted, it is stable at room temperature for 4 hours. Reconstituted product that has been prepared beyond this time limit should be discarded.

The TroVax® drug product is formulated in mannitol stabilizer and lyophilized, and is presented as a lyophilate, which is stored at ≤-15°C for a period of up to 12 months at the distributor's facilities, then is shipped at 2-8°C and is stable for up to 12 months further.

APPENDIX 2 Instructions for the Handling, Storage, Reconstitution and Disposal of TroVax®

TroVax® will be supplied by Oxford BioMedica U.K. Ltd.

The Investigator and the study site are responsible for investigational product accountability. All clinical study supplies that are delivered will be the responsibility of a suitably qualified and authorized person such as a hospital research pharmacist, who will document drug disposition and accountability for the duration of the trial.

Investigators and pharmacists should note that the clinical study supplies may only be used for the clinical study for which they are indicated. They must not be employed for any other study, whether of TroVax® or not, or for any other clinical use.

Additional information may be found in the current version of the Investigators Brochure.

Packaging and labelling

Packaging and labelling will be in accordance with Good Manufacturing Practice (GMP) for clinical trials.
Each vial will bear a label conforming to national regulations for an Investigational Medicinal Product.
The outer carton labelling will also bear a label conforming to national regulations for an Investigational Medicinal Product.

Storage and disposition of study medications

TroVax® must be stored in a locked refrigerator between 2°C to 8°C in the hospital pharmacy, or other comparable secure location. A temperature log should be maintained and monitored. TroVax® must be stored in such a way that it cannot be mixed up or confused with other medications, be they clinical trial supplies or medicines for routine clinical use.
Dispensing will be documented by completing a log with the date of dispensing and the subject details.

After notification from the sponsor all expired/unused/used vials will be destroyed on site in accordance with procedures for destruction of genetically modified waste and destruction will be documented appropriately.

Administration of TroVax®

TroVax® is administered at a dose of 1 x 10^9 $TCID_{50}$/mL in 1mL by intramuscular injection into the deltoid muscle of the upper arm. It is advised to alternate the arm in which TroVax® is injected at each cycle.

All subjects will receive the treatment in a side-room away from contact with other subjects. The formulation will be delivered to this side-room. TroVax® is presented as lyophilized material. Detailed instructions will be provided to the pharmacist for reconstitution. TroVax® must be re-suspended by adding 1.2 ml of water for injection. The resulting solution will appear opalescent. One ml volume of the solution is then withdrawn into a syringe and injected into the subject.

UNDER NO CIRCUMSTANCES MUST THE RECONSTITUTED MATERIAL BE ALLOWED TO STAND FOR MORE THAN FOUR HOURS AT ROOM TEMPERATURE. IF THIS DOES OCCUR, THE MATERIAL MUST BE DESTROYED AND A COMMENT MUST BE RECORDED IN THE PHARMACY FILE FOR ACCOUNTABILITY PURPOSES.

Handling and Disposal

Procedures for proper handling and disposal of anticancer drugs should conform to hospital procedures, guidelines and approved Biosafety SOPs. It is recommended to store used vials in labelled biohazard bags or disposed of by incineration or other such method approved by the local procedures only after drug accountability has been performed.

All healthcare staff handling TroVax® or materials contaminated by it must wear an apron, gloves, a mask and protective goggles. Pregnant nurses must not handle either TroVax® or materials contaminated by TroVax®.

Precautions/overdose

TroVax® is contraindicated in subjects who have previously had hypersensitive reactions to vaccinia vaccinations, egg proteins or to neomycin. In previous studies, the only consistent side effects of TroVax® were influenza-like symptoms and mild injection site reactions.

Subjects should remain under medical observation for one hour following injection with TroVax®. Adequate treatment provisions, including epinephrine injection (1:1000), should be available for immediate use should an anaphylactic reaction occur.

TroVax® is also contraindicated in subjects who are pregnant or lactating. Although highly unlikely, it is possible that an autoimmune response against the pituitary could occur since this organ stained positively for 5T4 in in vitro experiments. The Investigators should be alert to the possibility of pituitary insufficiency or failure.

No cases of TroVax® overdose have been reported. No active medical intervention is known to be required in the event of an overdose. The subject should be observed for as long as is considered appropriate by the Investigator based on the subject’s clinical condition and supportive care given if required.

APPENDIX 3 WHO Performance Status

0 - Asymptomatic (Fully active, able to carry on all predisease activities without restriction)

1 - Symptomatic but completely ambulatory (Restricted in physically strenuous activity but ambulatory and able to carry out work of a light or sedentary nature. For example, light housework, office work)

2 - Symptomatic, <50% in bed during the day (Ambulatory and capable of all self care but unable to carry out any work activities. Up and about more than 50% of waking hours)

3 - Symptomatic, >50% in bed, but not bedbound (Capable of only limited self-care, confined to bed or chair 50% or more of waking hours)

4 - Bedbound (Completely disabled. Cannot carry on any self-care. Totally confined to bed or chair)

5 - Death
