# Supplementary material for: Effect of Modified Vaccinia Ankara–5T4 and Low-Dose Cyclophosphamide on Antitumor Immunity in Metastatic Colorectal Cancer: A Randomized Clinical Trial
Source: JAMA Oncol. 2017 Sep 7;3(10):e172579. doi: 10.1001/jamaoncol.2017.2579 (PMC5824319; doi:10.1001/jamaoncol.2017.2579)
Supplement: Supplement 2. — eTable 1. Patient Baseline Characteristics eTable 2. Adverse Events eFigure 1. Effects of CPM and TroVax eFigure 2. Anti-MVA Antibody Responses Associated With Survival eFigure 3. Induced anti-5T4 Immunologic Responses Associated With Survival eFigure 4. PFS of TroVax-Treated or CPM-Treated Trial Groups eFigure 5. Tuberculin-PPD T-cell Response [file jamaoncol-3-524-s002.pdf]

Supplementary Online Content

Scurr M, Pembroke T, Bloom A, et al. Effect of modified vaccinia ankara–5T4 and low-dose cyclophosphamide on antitumor immunity in metastatic colorectal cancer: a randomized clinical trial. *JAMA Oncol*. Published online September 7, 2017. doi:10.1001/jamaoncol.2017.2579

eTable 1. Patient Baseline Characteristics

eTable 2. Adverse Events

eFigure 1. Effects of CPM and TroVax

eFigure 2. Anti-MVA Antibody Responses Associated With Survival

eFigure 3. Induced anti-5T4 Immunologic Responses Associated With Survival

eFigure 4. PFS of TroVax-Treated or CPM-Treated Trial Groups

eFigure 5. Tuberculin-PPD T-cell Response

© 2017 Scurr M et al. *JAMA Oncology*

eTable 1. Patient Baseline Characteristics

Characteristic	Group 1 (n=8)	Group 2 (n=9)	Group 3 (n=17)	Group 4 (n=18)
Sex				
Female	2 (25%)	5 (56%)	3 (18%)	4 (22%)
Male	6 (75%)	4 (44%)	14 (82%)	14 (78%)
Mean age (years)	62.5	64.6	63.4	65.5
Site of Primary				
Right	1 (13%)	4 (44%)	3 (18%)	1 (6%)
Left	4 (50%)	3 (33%)	8 (47%)	6 (33%)
Sigmoid / Rectum	3 (38%)	2 (22%)	6 (35%)	8 (44%)
Not identified	0 (0%)	0 (0%)	0 (0%)	3 (17%)
Site of Metastases				
Liver	6 (75%)	5 (56%)	8 (47%)	14 (78%)
Lung	4 (50%)	4 (44%)	9 (53%)	7 (39%)
Peritoneum	5 (63%)	4 (44%)	3 (18%)	7 (39%)
WHO performance Status				
0	4 (50%)	5 (56%)	6 (35%)	10 (56%)
1	4 (50%)	4 (44%)	10 (59%)	8 (44%)
2	0 (0%)	0 (0%)	1 (6%)	0 (0%)
Previous treatment				
Primary colectomy	7 (88%)	3 (33%)	10 (59%)	12 (67%)
Capecitabine	8 (100%)	9 (100%)	17 (100%)	18 (100%)
5-Fluorouracil	8 (100%)	8 (89%)	16 (94%)	16 (89%)
Irinotecan	4 (50%)	3 (33%)	7 (41%)	8 (44%)
Oxaliplatin	3 (38%)	4 (44%)	6 (35%)	5 (28%)
Cetuximab	0 (0%)	0 (0%)	0 (0%)	3 (17%)
Aflibercept	0 (0%)	1 (11%)	1 (6%)	1 (6%)

© 2017 Scurr M et al. *JAMA Oncology*

eTable 2. Adverse Events

	Trial Group 1 (n=8)			Trial Group 2 (n=9)			Trial Group 3 (n=17)			Trial Group 4 (n=18)		
	Grade 1-2	Grade 3	Grade 4	Grade 1-2	Grade 3	Grade 4	Grade 1-2	Grade 3	Grade 4	Grade 1-2	Grade 3	Grade 4
Any serious adverse event	0 (0%)	0 (0%)	0 (0%)	0 (0%)	0 (0%)	0 (0%)	0 (0%)	0 (0%)	2 (12%)	0 (0%)	0 (0%)	2 (11%)
Any adverse event	4 (50%)	0 (0%)	0 (0%)	7 (78%)	0 (0%)	0 (0%)	13 (76%)	1 (6%)	2 (12%)	16 (89%)	1 (6%)	2 (11%)
Event												
Fatigue	0 (0%)	0 (0%)	0 (0%)	1 (11%)	0 (0%)	0 (0%)	0 (0%)	0 (0%)	0 (0%)	2 (11%)	0 (0%)	0 (0%)
Nausea and vomiting	1 (13%)	0 (0%)	0 (0%)	0 (0%)	0 (0%)	0 (0%)	2 (12%)	0 (0%)	0 (0%)	6 (33%)	0 (0%)	1 (6%)
Injection-site reaction	0 (0%)	0 (0%)	0 (0%)	0 (0%)	0 (0%)	0 (0%)	7 (41%)	0 (0%)	0 (0%)	5 (28%)	0 (0%)	0 (0%)
Diarrhea	0 (0%)	0 (0%)	0 (0%)	1 (11%)	0 (0%)	0 (0%)	0 (0%)	0 (0%)	0 (0%)	1 (6%)	0 (0%)	0 (0%)
Constipation	0 (0%)	0 (0%)	0 (0%)	1 (11%)	0 (0%)	0 (0%)	2 (12%)	0 (0%)	0 (0%)	2 (11%)	0 (0%)	0 (0%)
Myalgia / arthralgia	2 (25%)	0 (0%)	0 (0%)	2 (22%)	0 (0%)	0 (0%)	2 (12%)	0 (0%)	0 (0%)	5 (28%)	0 (0%)	0 (0%)
Hematochezia	0 (0%)	0 (0%)	0 (0%)	0 (0%)	0 (0%)	0 (0%)	0 (0%)	0 (0%)	0 (0%)	2 (11%)	0 (0%)	0 (0%)
Headache	0 (0%)	0 (0%)	0 (0%)	1 (11%)	0 (0%)	0 (0%)	1 (6%)	0 (0%)	0 (0%)	1 (6%)	0 (0%)	0 (0%)
Abdominal pain	0 (0%)	0 (0%)	0 (0%)	0 (0%)	0 (0%)	0 (0%)	1 (6%)	1 (6%)	0 (0%)	2 (11%)	1 (6%)	1 (6%)
Dizziness	0 (0%)	0 (0%)	0 (0%)	0 (0%)	0 (0%)	0 (0%)	0 (0%)	0 (0%)	0 (0%)	2 (11%)	0 (0%)	0 (0%)
Erythema / rash	0 (0%)	0 (0%)	0 (0%)	1 (11%)	0 (0%)	0 (0%)	2 (12%)	0 (0%)	0 (0%)	0 (0%)	0 (0%)	0 (0%)
Respiratory tract infection	1 (13%)	0 (0%)	0 (0%)	2 (22%)	0 (0%)	0 (0%)	7 (41%)	0 (0%)	0 (0%)	2 (11%)	0 (0%)	0 (0%)
Thromboembolism	0 (0%)	0 (0%)	0 (0%)	0 (0%)	0 (0%)	0 (0%)	1 (6%)	0 (0%)	0 (0%)	0 (0%)	0 (0%)	0 (0%)
Aphthous ulcer	0 (0%)	0 (0%)	0 (0%)	0 (0%)	0 (0%)	0 (0%)	0 (0%)	0 (0%)	0 (0%)	1 (6%)	0 (0%)	0 (0%)
Urinary tract infection	0 (0%)	0 (0%)	0 (0%)	0 (0%)	0 (0%)	0 (0%)	1 (6%)	0 (0%)	0 (0%)	0 (0%)	0 (0%)	0 (0%)
GI hemorrhage	0 (0%)	0 (0%)	0 (0%)	0 (0%)	0 (0%)	0 (0%)	0 (0%)	0 (0%)	1 (6%)	0 (0%)	0 (0%)	0 (0%)
Raised ALP	0 (0%)	0 (0%)	0 (0%)	0 (0%)	0 (0%)	0 (0%)	1 (6%)	0 (0%)	0 (0%)	0 (0%)	0 (0%)	0 (0%)
Anemia	0 (0%)	0 (0%)	0 (0%)	0 (0%)	0 (0%)	0 (0%)	0 (0%)	0 (0%)	0 (0%)	2 (11%)	0 (0%)	0 (0%)
Atrial fibrillation	0 (0%)	0 (0%)	0 (0%)	0 (0%)	0 (0%)	0 (0%)	0 (0%)	0 (0%)	0 (0%)	1 (6%)	0 (0%)	0 (0%)
Transient ischemic attack	0 (0%)	0 (0%)	0 (0%)	0 (0%)	0 (0%)	0 (0%)	1 (6%)	0 (0%)	0 (0%)	0 (0%)	0 (0%)	0 (0%)
Parasthesia	0 (0%)	0 (0%)	0 (0%)	0 (0%)	0 (0%)	0 (0%)	0 (0%)	0 (0%)	0 (0%)	1 (6%)	0 (0%)	0 (0%)
Stoma site pain	0 (0%)	0 (0%)	0 (0%)	0 (0%)	0 (0%)	0 (0%)	0 (0%)	0 (0%)	0 (0%)	1 (6%)	0 (0%)	0 (0%)
Fever	0 (0%)	0 (0%)	0 (0%)	0 (0%)	0 (0%)	0 (0%)	0 (0%)	0 (0%)	1 (6%)	0 (0%)	0 (0%)	0 (0%)

Other than injection site reactions, no evidence that any adverse events were treatment related. Patients with adverse events in more than one category are counted separately in each group.

© 2017 Scurr M et al. *JAMA Oncology*

eFigure 1. Effects of CPM and TroVax

A.

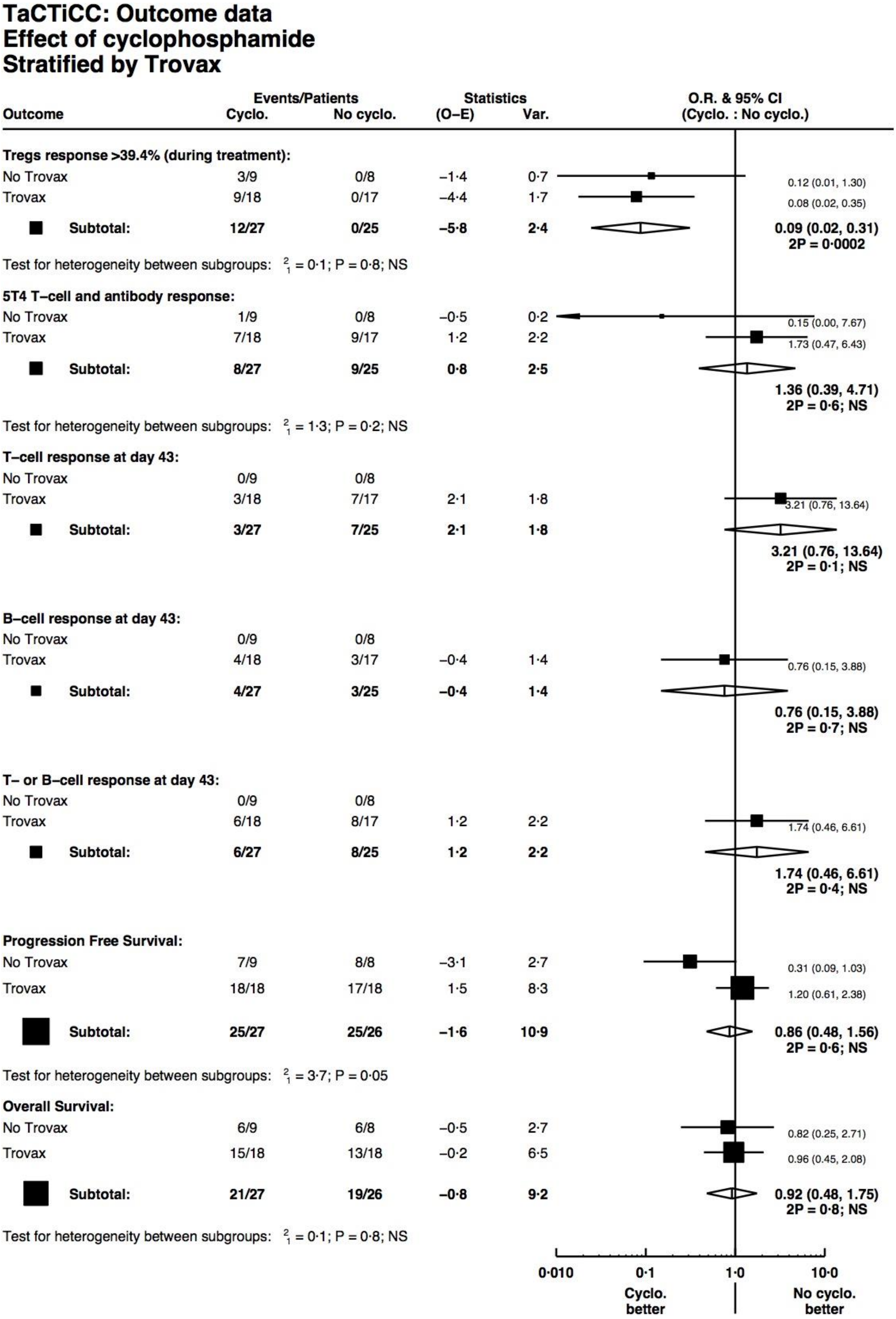

© 2017 Scurr M et al. *JAMA Oncology*

B.

TaCTiCC: Outcome data
Effect of Trovax
Stratified by Cyclophosphamide

Outcome	Events/Patients Trovax	Events/Patients No Trovax	Statistics (O–E)	Statistics Var.	O.R. & 95% CI (Trovax : No Trovax)
**Tregs response >39.4%:**					
No Cyclophosphamide	0/17	0/8			
Cyclophosphamide	9/18	3/9	−1·0	1·5	0.52 (0.11, 2.53)
■ **Subtotal:**	**9/35**	**3/17**	**−1·0**	**1·5**	**0.52 (0.11, 2.53)** **2P = 0·4; NS**
**5T4 T−cell and antibody response:**					
No Cyclophosphamide	9/17	0/8	−2·9	1·3	0.11 (0.02, 0.61)
Cyclophosphamide	7/18	1/9	−1·7	1·3	0.28 (0.05, 1.55)
■ **Subtotal:**	**16/35**	**1/17**	**−4·5**	**2·6**	**0.17 (0.05, 0.59)** **2P = 0·005**
Test for heterogeneity between subgroups: $^{2}_{1}$ = 0·6; P = 0·5; NS					
**T−cell response at day 43:**					
No Cyclophosphamide	7/17	0/8	−2·2	1·1	0.14 (0.02, 0.88)
Cyclophosphamide	3/18	0/9	−1·0	0·6	0.20 (0.02, 2.40)
■ **Subtotal:**	**10/35**	**0/17**	**−3·2**	**1·8**	**0.16 (0.04, 0.69)** **2P = 0·01**
Test for heterogeneity between subgroups: $^{2}_{1}$ = 0·0; P = 0·8; NS					
**B−cell response at day 43:**					
No Cyclophosphamide	3/17	0/8	−1·0	0·6	0.20 (0.02, 2.53)
Cyclophosphamide	4/18	0/9	−1·3	0·8	0.18 (0.02, 1.67)
■ **Subtotal:**	**7/35**	**0/17**	**−2·3**	**1·4**	**0.19 (0.04, 1.01)** **2P = 0·05**
Test for heterogeneity between subgroups: $^{2}_{1}$ = 0·0; P = 1·0; NS					
**T− or B−cell response at day 43:**					
No Cyclophosphamide	8/18	0/8	−2·5	1·2	0.13 (0.02, 0.79)
Cyclophosphamide	6/18	0/9	−2·0	1·1	0.16 (0.02, 1.03)
■ **Subtotal:**	**14/36**	**0/17**	**−4·5**	**2·3**	**0.14 (0.04, 0.52)** **2P = 0·003**
Test for heterogeneity between subgroups: $^{2}_{1}$ = 0·0; P = 0·9; NS					
**Progression Free Survival:**					
No Cyclophosphamide	17/18	8/8	−3·6	3·0	0.31 (0.10, 0.94)
Cyclophosphamide	18/18	7/9	3·5	5·4	1.90 (0.82, 4.43)
■ **Subtotal:**	**35/36**	**15/17**	**−0·1**	**8·4**	**0.98 (0.50, 1.93)** **2P = 1·0; NS**
Test for heterogeneity between subgroups: $^{2}_{1}$ = 6·5; P = 0·01					
**Overall Survival:**					
No Cyclophosphamide	13/18	6/8	0·4	3·9	1.11 (0.41, 2.99)
Cyclophosphamide	15/18	6/9	0·9	4·5	1.21 (0.48, 3.07)
■ **Subtotal:**	**28/36**	**12/17**	**1·3**	**8·4**	**1.16 (0.59, 2.29)**

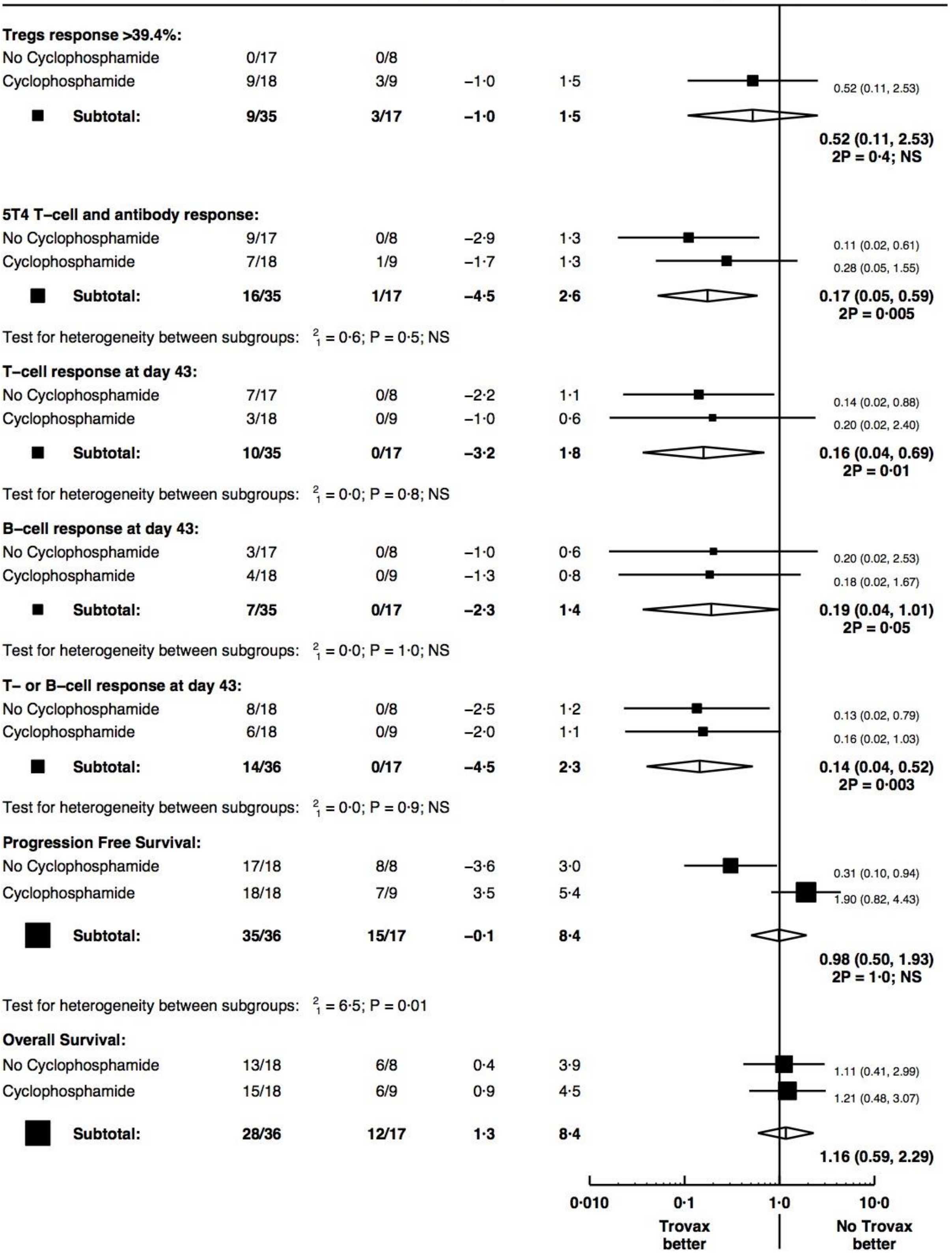

© 2017 Scurr M et al. *JAMA Oncology*

eFigure 2. Anti-MVA Antibody Responses Associated With Survival

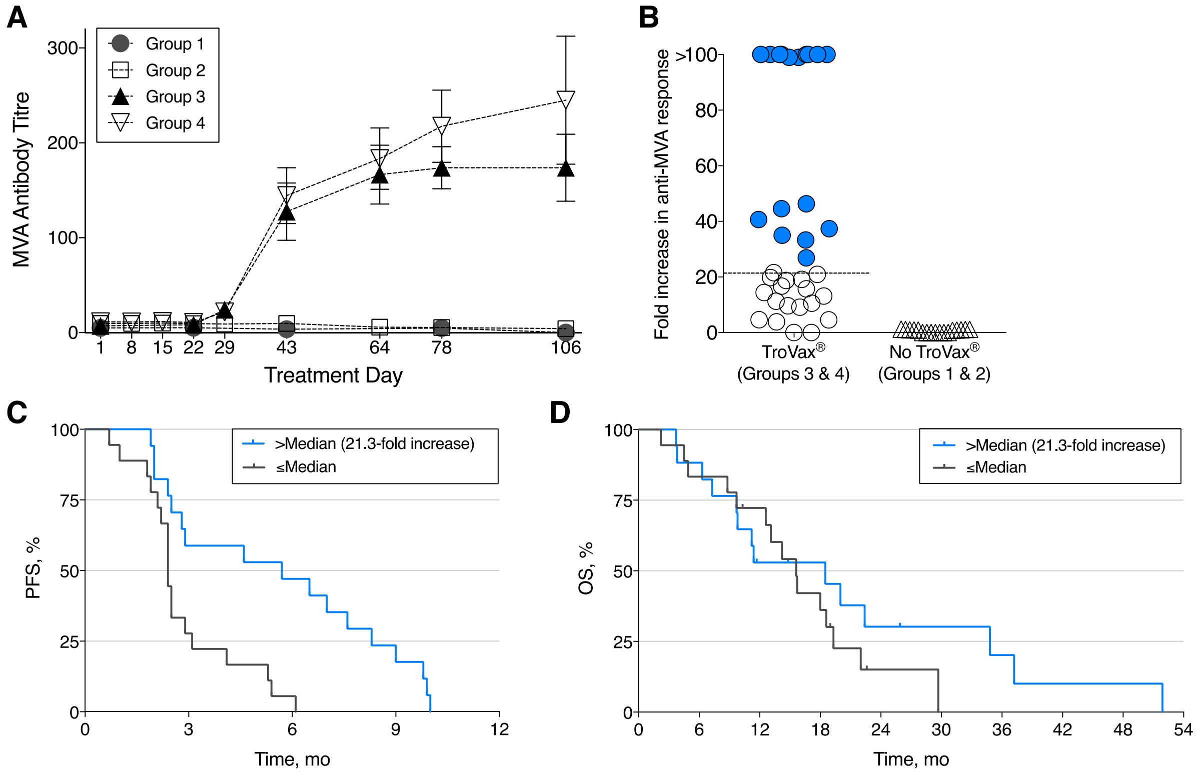

No. at risk

>21.6-fold ↑ in MVA titre	17	10	8	3	1	17	15	8	7	4	3	2	1	1
≤21.6-fold ↑ in MVA titre	18	5	1	0	0	18	15	12	6	1	0	0	0	0

(A) MVA-specific antibody titres were measured from plasma samples taken throughout the course of the trial. (B) The fold increases in MVA-specific antibody responses were calculated by dividing the highest response to TroVax® at TD29-106 by baseline level at TD1. TroVax® recipients (groups 3 & 4) demonstrating a >21.3-fold increase in anti-MVA antibody responses are highlighted in blue (n=17). These responses were associated with PFS (C: HR 0.26 (95% CI 0.11-0.59); p=.001) and OS (D: HR 0.69 (95% CI 0.32-1.51); p=.36).

© 2017 Scurr M et al. *JAMA Oncology*

eFigure 3. Induced Anti-5T4 Immunologic Responses Associated With Survival

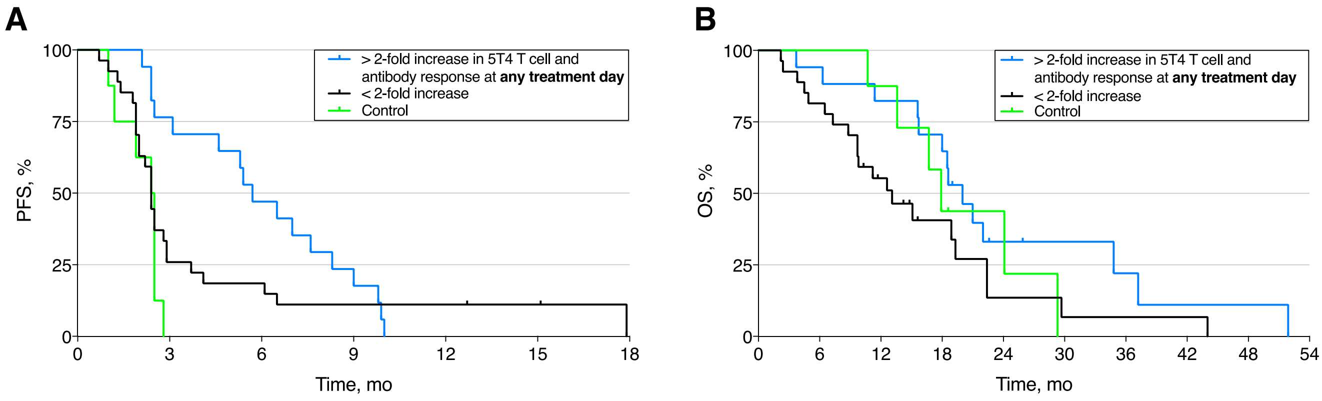

No. at risk

	A						B								
Doubled 5T4 response	17	13	8	3	0	0	17	16	14	11	4	3	2	1	1
Poor / No response	27	7	5	3	3	2	27	22	13	6	2	1	1	1	0
Control	8	0	0	0	0	0	8	8	6	3	2	0	0	0	0

The fold increases in 5T4-specific IFN-γ^+ T-cell and antibody responses were calculated by dividing the highest response to treatment at TD8-106 by baseline (TD1) level. Patients of any treatment group demonstrating a >2-fold increase in both anti-5T4 T-cell and antibody responses are highlighted in blue (n=17), and were associated with PFS (A: HR (treated patients) 0.58 (95% CI 0.31-1.09); p=.09) and OS (B: HR (treated patients) 0.56 (95% CI 0.28-1.12); p=.1).

© 2017 Scurr M et al. *JAMA Oncology*

eFigure 4. PFS of TroVax-Treated or CPM-Treated Trial Groups

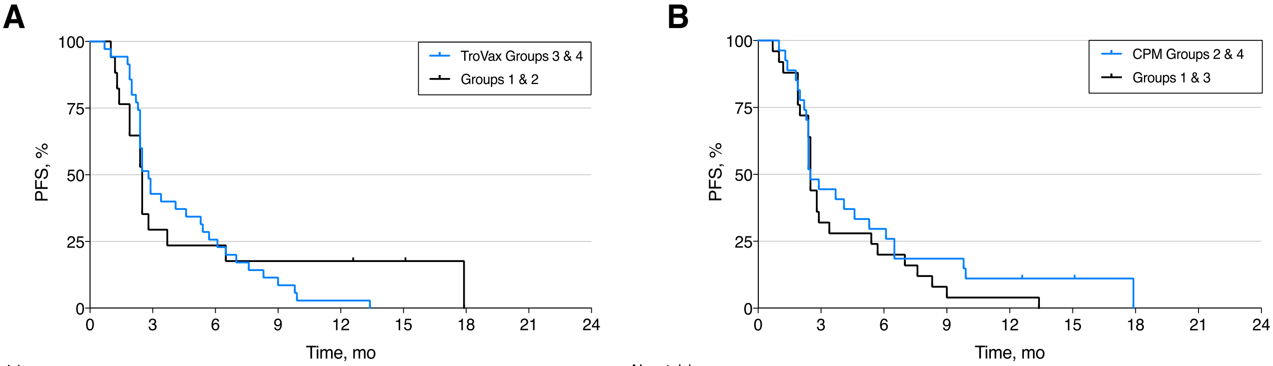

A

No. at risk

	0	3	6	9	12	15	18	21
Groups 3 & 4	35	15	9	3	1	0	0	0
Groups 1 & 2	17	5	4	3	3	2	0	0

B

No. at risk

	0	3	6	9	12	15	18	21
Groups 2 & 4	27	12	8	5	3	2	0	0
Groups 1 & 3	25	8	5	1	1	0	0	0

PFS of all patients receiving TroVax® (groups 3 and 4, n=35) vs. no TroVax® (groups 1 and 2, n=17) is indicated (A: HR 1.11 (95% CI 0.63-2.16); p=.6). PFS of all patients receiving CPM (groups 2 and 4, n=27) vs. no CPM (groups 1 and 3, n=25) (B: HR 0.72 (95% CI 0.40-1.28); p=.3) is indicated.

© 2017 Scurr M et al. *JAMA Oncology*

eFigure 5. Tuberculin-PDD T-cell Response

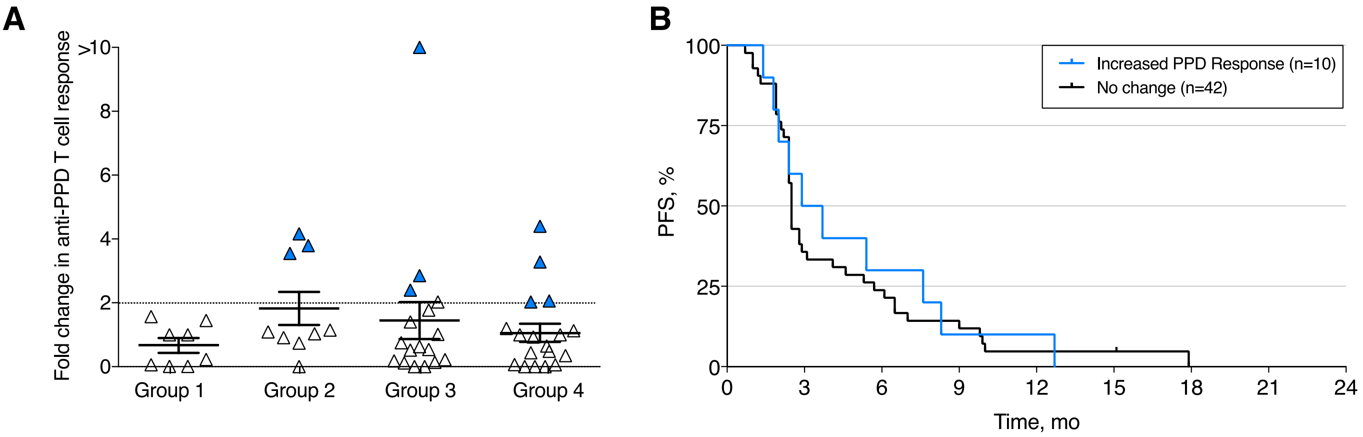

No. at risk							
PPD increase	10	12	8	5	3	2	0
No change	42	8	5	1	1	0	0

Tuberculin-PPD T-cell responses remain stable during the trial, and do not associate with PFS. Tuberculin-PPD T-cell responses were monitored by IFN-γ ELISPOT throughout the course of the trial. Fold changes in IFN-γ^{+} PPD-specific responses are indicated (A). Those patients exhibiting increased anti-PPD responses during the trial are highlighted in blue (n=10) and were associated with PFS (B: HR 0.90 (95% CI 0.45-1.80); p=.76).

© 2017 Scurr M et al. *JAMA Oncology*
